# Genetic and phylogeographic evidence for Jewish Holocaust victims at the Sobibór death camp

**DOI:** 10.1186/s13059-021-02420-0

**Published:** 2021-08-06

**Authors:** Marta Diepenbroek, Christina Amory, Harald Niederstätter, Bettina Zimmermann, Maria Szargut, Grażyna Zielińska, Arne Dür, Iwona Teul, Wojciech Mazurek, Krzysztof Persak, Andrzej Ossowski, Walther Parson

**Affiliations:** 1 Department of Forensic Genetics, Pomeranian Medical University, Szczecin, Poland; 2 Institute of Legal Medicine, Ludwig Maximilian University of Munich, Munich, Germany; 3 Institute of Legal Medicine, Medical University of Innsbruck, Innsbruck, Austria; 4 Institute of Mathematics, University of Innsbruck, Innsbruck, Austria; 5 Institute of Anatomy, Pomeranian Medical University, Szczecin, Poland; 6 “Sub Terra” Badania Archeologiczne, Chełm, Poland; 7 Institute of Political Studies, Polish Academy of Sciences, Warsaw, Poland; 8 Forensic Science Program, The Pennsylvania State University, University Park, PA USA

## Abstract

Six million Jews were killed by Nazi Germany and its collaborators during World War II. Archaeological excavations in the area of the death camp in Sobibór, Poland, revealed ten sets of human skeletal remains presumptively assigned to Polish victims of the totalitarian regimes. However, their genetic analyses indicate that the remains are of Ashkenazi Jews murdered as part of the mass extermination of European Jews by the Nazi regime and not of otherwise hypothesised non-Jewish partisan combatants. In accordance with traditional Jewish rite, the remains were reburied in the presence of a Rabbi at the place of their discovery.

**Supplementary Information:**

The online version contains supplementary material available at 10.1186/s13059-021-02420-0.

## Introduction

In Nazi parlance, the euphemism “final solution of the Jewish problem” (*die Endlösung der Judenfrage*) denoted the idea to exterminate all Jews of Europe. The persecution of Jews begun immediately after Hitler’s rise to power. It transformed through a stage of the policy of forced emigration in the late 1930s into the mass scale murder of six million Jews and other persecuted groups during the Second World War [1, 2]. The secret Operation Reinhardt (OR), which was launched in March 1942, was one of the deadliest contributions to this process. By December 1942, the lives of 1.3 million people were taken in gas chambers at death camps in Bełżec, Sobibór and Treblinka, with many thousands more killed on the spot during the liquidation of the ghettos in German-occupied Poland. The total death toll of OR amounts to nearly two million. A recently published study concludes that more than 25% of all Holocaust victims were killed during only 3 months [2]. In contrast to the concentration camps in Auschwitz-Birkenau and Majdanek, the three OR death camps were not designed as labour camps, but rather exclusively served the purpose of industrial scale mass murder [1]. After having fulfilled this purpose, Bełżec, Sobibór and Treblinka were closed and their traces scrupulously obliterated (Additional File 1).

The level of secrecy of OR is unparalleled. Evidence supporting the functioning of the extermination camp in Sobibór is scarce and only based on a few testimonies of Ukrainian and German guards, as well as a few prisoners who succeeded in escaping from the camp [1]. Archaeological investigations were initiated in 2000 in the area where the extermination camp was reported in Sobibór, to shed more light onto OR. The fieldwork revealed pits that were interpreted as locations to bury bodies and/or their burnt remains [1]. Later archaeological work in 2013 led to the discovery of remaining walls of gas chambers, which was suggested as tangible proof of criminal activity [1]. However, the most surprising discovery was the detection of ten almost fully intact male skeletons (Additional File 1: Table S1, Fig. S1-S4). This was particularly surprising, as the testimonies suggested complete cremation of all Jewish victims in Sobibór [1]. Based on the archaeological analysis of the burials and information gathered by local historians, it was initially assumed that these remains may have belonged to a group of Polish partisans, who were killed in the 1950s by the communist government and buried secretly in that area. A local branch of the Institute of National Remembrance, based in Lublin, initiated an interdisciplinary investigation towards the identification of members of the anti-communist underground movement. According to the primary assumption regarding the origin of the remains, they were further investigated at the Department of Forensic Genetics, Pomeranian Medical University in Szczecin. This institute curates the Polish Genetic Database of Victims of Totalitarianisms (https://www.pbgot.pl), with the purpose of employing forensic state-of-the-art methods to identify victims of Communist and Nazi totalitarian regimes in Poland [3]. The forensic investigations revealed unquestionable gunshot traces, which affected five individuals. Four of the shots were headshots, which are interpreted as the result of an execution (Additional File 1: Fig. S5). The artefacts were mostly damaged and corroded. The best-preserved items were described as personal belongings, however without any specific features that would enable their dating. The most significant findings were bullet casings that could be assigned to a type of weapon known to be part of the standard equipment of German guards in concentration and extermination camps [1] (Additional File 1: Fig. S6-S8).

## Results and discussion

Molecular genetic analyses on DNA extracted from the human remains were performed independently in two forensic genetic laboratories. The extracts yielded only low amounts of mitochondrial DNA (mtDNA), ranging between ~ 70 to ~ 4000 copies/μl (Additional File 1: Table S2), which roughly corresponds to less than up to 16 cell equivalents of nuclear DNA (assuming 250 copies mtDNA/cell). Nevertheless, the genetic analyses yielded confirmatory mtDNA and Y-chromosomal DNA haplotypes.

**Fig. 1:**
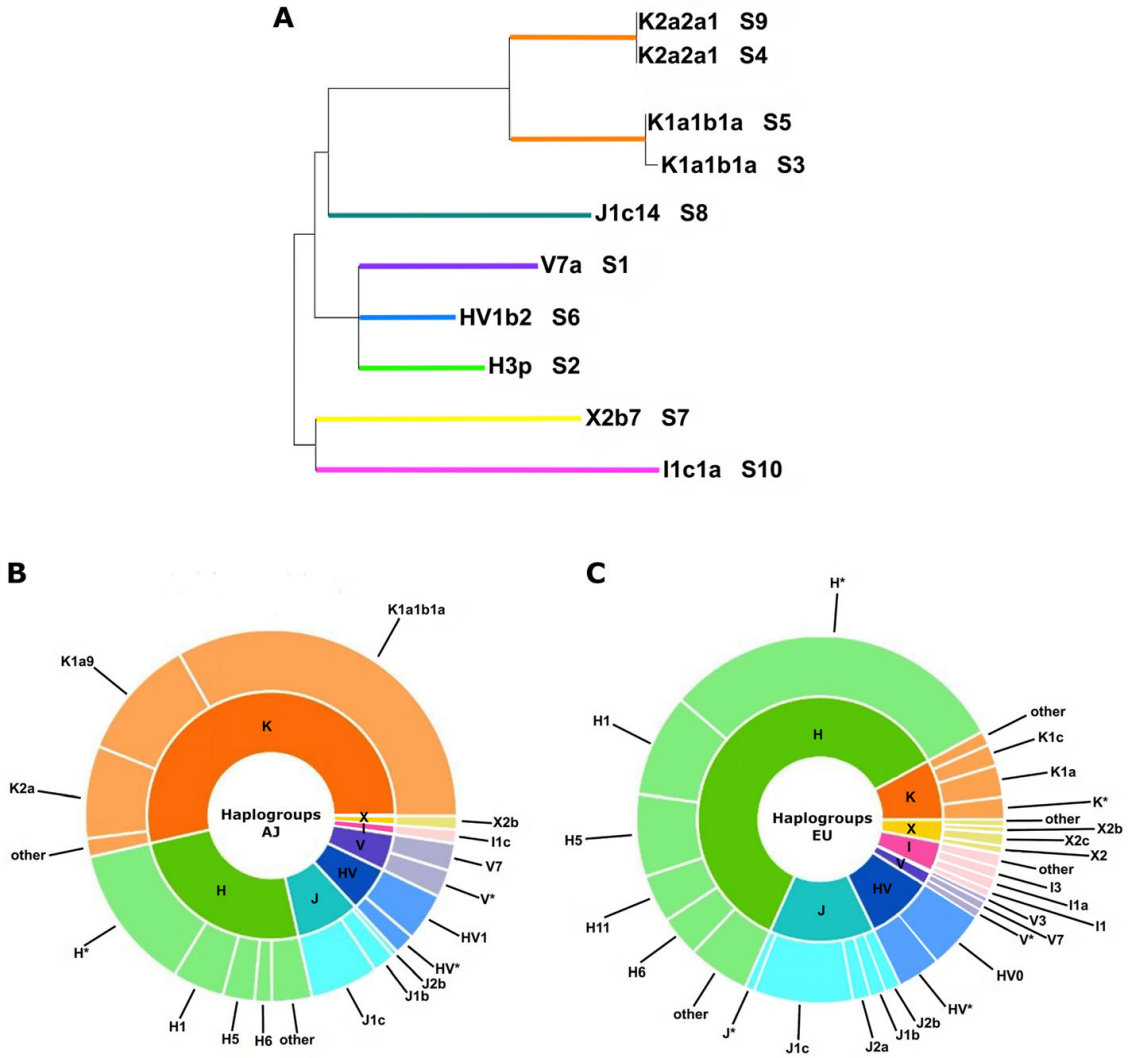
Mitochondrial DNA haplogroups of the ten studied skeletal remains (S1-10) and their frequencies amongst Ashkenazi Jews (AJ) and other Europeans. **A** Representation of the estimated haplogroups (https://empop.online; 13, 14) on a Maximum Likelihood tree created in MEGA X [49–52]. **B** Relative frequencies of the estimated control region haplogroups amongst AJ of different European origin [4–8]. **C** Relative frequencies of the estimated control region haplogroups amongst corresponding European populations without AJ samples [13, 14]

**Fig. 2:**
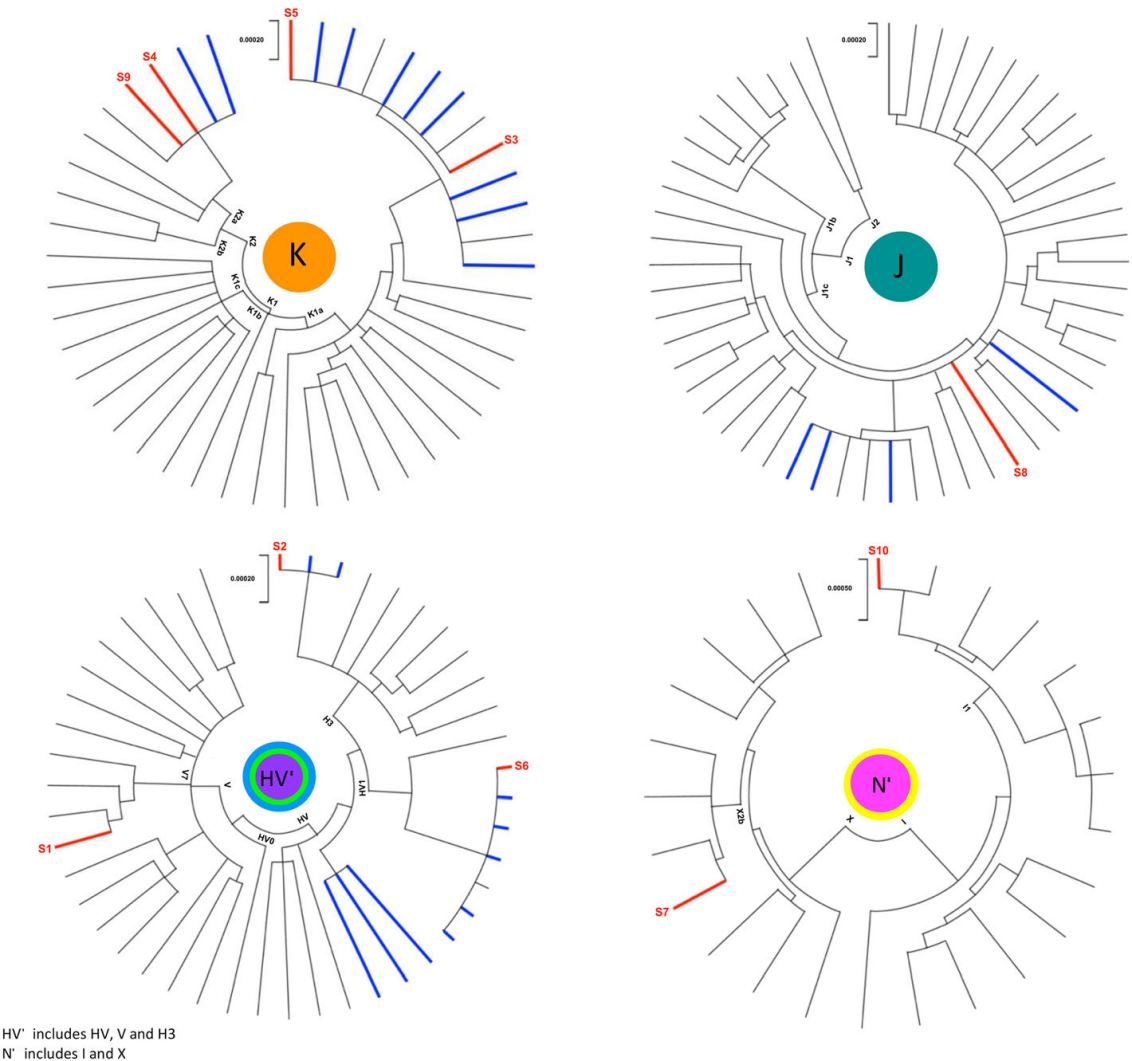
Mitochondrial DNA haplogroup distribution in Poles (black), modern AJ (blue) and the studied remains (red). Each branch corresponds to one single individual. The dataset included 136 Polish and 24 Jewish mitogenomes acquired from GenBank (Additional File 1: Table S6). The evolutionary history was inferred by using the Maximum Likelihood method and Tamura 3-parameter model. Evolutionary analyses were conducted in MEGA X [49–52]

Full mitogenome sequences were obtained for all ten sets of remains (Additional File 1: Table S3). The sequences were assigned to haplogroups K1a1b1a (two individuals), K2a2a1 (two individuals), H3p, HV1b2, I1c1a, J1c14, V7a and X2b7 (each belonging to a single individual, Fig. 1A). We note that the remains S4 and S9 share the same mitogenome and could thus be maternally related. Eight of the ten control region (CR) mitotypes resulted in full matches in the mtDNA database EMPOP (https://empop.online, R13 (N = 38,361)) [13, 14]. All except one of those matches consisted of, or at least included, samples of known Ashkenazi origin (Additional File 1: Table S4). The mitotypes were also evaluated by contrasting the probabilities of the studied haplotypes in two populations of interest, namely Poles and modern Ashkenazi Jews (AJ). All ten haplotypes of the remains resulted in higher probability values in the AJ dataset (Additional File 1: Table S5). This led to the formulation of an alternative hypothesis on the ancestry of the individuals and put a possible Ashkenazim origin in the focus. The largest published Ashkenazim mtDNA datasets are based on CR sequences [4–8]. We used those sequences to estimate relative frequencies of the haplogroups observed amongst AJ of different European origin (Fig. 1B) and contrasted them to the distribution of the same haplogroups amongst corresponding European populations (Fig. 1C). Both our data and data from literature show that all haplogroups established for the studied individuals correspond to lineages found in Ashkenazim, while being rare or missing in other European populations [5, 6, 9–12]. According to earlier studies, the ancestors of modern AJ are considered to be mainly of Central and Eastern European descent [9], with some evidence also pointing towards the Near East. It was established that approximately 40% of modern Ashkenazim belong to the four haplogroups K1a1b1a, K1a9, K2a2a, and N1b, which are otherwise infrequent amongst European and Near Eastern populations [5, 6, 9, 10]. The comparative search was also performed for the mitogenome data obtained for the ten sets of remains, querying a vetted dataset of 27,373 worldwide mitogenomes downloaded from GenBank. Full matches were found for nine of the ten mitotypes with all but one including mitogenomes of known Ashkenazi origin (Additional File 1: Table S6). In view of the initial hypothesis that the remains might belong to Polish partisans, further phylogeographic analyses were performed on a selected set of representative mitotypes observed in Poland (*N* = 540; Additional File 1: Table S6) [15, 16] and on available AJ mitogenomes. These analyses revealed that four of the established haplogroups clustered with known Ashkenazi lineages (Fig. 2).

Y-chromosomal Short Tandem Repeat (STR) analyses resulted in five full and five partial profiles, of which three were almost complete (Additional File 1: Table S7). The Y-haplogroup estimation (Nevgen; https://www.nevgen.org) assigned eight of the ten studied individuals to haplogroups G, E and J, which was confirmed by SNP sequencing (Additional File 1: Table S8). Compared to other modern European populations, these haplogroups are more abundant in AJ [17–19]. The two remaining Y chromosomes were assigned to two haplogroup R lineages, which are frequently observed in both AJ [20] and other European populations. Most of the published studies suggest that the Y-chromosomal lineages found amongst Ashkenazim originate in the Near East, with some possible gene flow from the host European populations [20, 21]. Some genetic studies involving modern Jews focused on male descendants from the Tribe of Levi, who served particular religious duties for the Israelites. The results revealed that over 50% of the studied individuals belong to haplogroup R1a [20] and further studies suggested that the R-M582 lineage is rare in non-Jewish populations, while being common in Ashkenazi Levites and other Ashkenazi males [22, 23].

Four of the studied individuals belonged to the J-P58 lineage, which, in Europe, is found almost exclusively amongst AJ [17, 18]. These studies also indicated that this haplogroup is prevalent in the Jewish priesthood, Cohanim. Cohanim are believed to be the direct patrilineal descendants of the biblical Aaron, and it is traditionally accepted that only men with this ancestry can belong to the Jewish Cohanim. Already in the late 1990s, it was discovered that this group mostly represents the same Y-chromosomal haplotype [24, 25]. The first published so-called Cohen Modal Haplotypes (CMH) were based on six Y-STR markers [25]. This set was later extended to 12 Y-STR loci [18]. The remains studied here were analysed with 27 Y-STR markers, including 10 of the 12 CMH loci. The remaining two CMH markers, DYS388 and DYS426, were sequenced in independent assays. Two of the studied individuals showed a full match with the CMH (14-16-23-10-11-12-13/15-13-30-11-12) and the remaining two revealed close neighbours (14-15-23-10-11-12-13/15-13-30-11-13; 14-16-23-10-11-12-13/17-13-31-11-11).

## Conclusions

The analyses of both the maternal and the paternal heritage of the ten unknown remains resulted for all the individuals in haplotypes that were at least very similar and, in some cases, even identical to the lineages observed in AJ. These genetic results, in combination with the non-genetic findings, such as artefact analysis and anthropological assessment, provide strong evidence that the Sobibór remains point at a Jewish origin of the victims, rather than the alternatively hypothesised European origin. In the light of these results, the prosecutor of the Institute of National Remembrance in Lublin, who incited the identification process, ordered the reburial of the remains. Following Jewish rite, the ceremony was led by a Rabbi and the victims were buried in separate graves at the places of their discovery.

## Methods

### Archaeological work carried out in the former extermination camp in Sobibór

Systematic archaeological research began in 2000 and continued in 2001, when a group from the Institute of Archaeology and Ethnology at the Nicolaus Copernicus University in Toruń, led by Prof. Andrzej Kola, performed a series of bore-holes in search of the location of mass graves. As a result of these, seven mass graves, with the remains of camp victims, as well as five archaeological sites, located south of the mass graves, were discovered. One of them has been unambiguously determined as a relic of gas chambers. In 2004, non-invasive geophysical surveys were performed and from 2007 to 2010 systematic excavations were carried out. As a result, the first relics of *Lager III*/Camp III were discovered. Extensive excavation research, which was related to the planned commemoration of the victims of the camp, was carried out from 2011 to 2016.

During the archaeological research, 22 graves were discovered. All were located within the area of *Lager III*/Camp III. Graves numbered 1–7 and 8/15 (Additional File 1: Fig. S1) were mass graves of cremation nature in the upper layer and of skeletal nature in the lower, where the remains of the victims showed signs of grave wax. Graves 9–11 and 17–20 were small cremation graves, perhaps containing remains of Jewish prisoners from Treblinka, who were brought to Sobibór to liquidate the camp after the decision to close it. Grave 21 was an archaeological site, discovered in the autumn of 2016 near the *Sonderkommando* barracks, carrying numerous teeth and dental crowns, which were probably removed from the victims just after the gassings. The remaining graves were untouched skeletal burials. A total of ten skeletons, buried in four graves [12–14 and 16], were found. All of them were discovered in 2013. In 2017, during the supervision of construction works, grave number 22 was discovered. It was located outside the northern fence of *Lager III*/Camp III. Mixed bone remains of at least three people were discovered in the grave. Due to a Rabbi’s representative decision based on Jewish law, these remains were left untouched and reburied.

The first two skeletal graves were discovered in autumn 2012, during the excavation of mass graves in order to verify their function. Initially, those archaeological sites showed an undefined function and they seemed to be dug in the north-western corner of the original fence of *Lager III*/Camp III. The continuation of exploration of these objects in spring of 2013 led to the unveiling of grave 12, in which the remains of six people were found, as well as of grave 13, in which one person was buried. The local conservator’s office, the police and the District Prosecutor’s Office were notified of the discovery of these graves, which initiated further investigation into this matter. The representative of the Prosecutor’s Office authorised the documentation of human remains in graves 12 and 13 in accordance with archaeological methodology, which allowed full and optimal documentation of the discovered human remains at the place of discovery. The results of this documentation, along with a list of artefacts, recognised as evidence, were forwarded to the Prosecutor’s Office for further investigation. Based on the character of the discovery, the case was taken over by the Institute of National Remembrance in Lublin at the end of 2013. Initially, two possible hypotheses were articulated as to whom those remains may belong. One connected the burials with the executions of the last prisoners from Treblinka, who came to Sobibór to liquidate the camp in 1943 (hypothesis rejected). The second hypothesis explained the rush of the execution and burying the bodies by connecting it to the activities of The People’s Commissariat for Internal Affairs (abbreviated NKVD), which was the interior ministry of the Soviet Union. There was a report on NKVD activity after the war in the area of the former camp. Residents of the villages in the area surrounding the extermination camp witnessed shots. The archaeological analyses of the burial sites suggested that the graves were rather connected to the crimes committed by NKVD between 1945 and 1956. As a significant observation, the layers of the burial pits were crossing the camp ground layers, namely dark sand (quite probably coming from time when the death camp was operating in 1942–1943) and grey sand (containing many burnt, crushed human bones). This last layer was most likely created as a result of the removal of remnants of burnt human remains in the area of above-ground crematoria, located near skeletal graves 14 and 16. This can be most likely dated to the time when the camp was closed in autumn 1943. These observations suggest that the execution of people, whose remains were discovered in grave 16, but also in graves 12–14, occurred after the end of the criminal activity of the German Nazi extermination camp.

In grave 12, which measured 1.90 by 1.90 m and was 0.80 m deep, six people were buried. Grave 13, which was located near grave 12, was much shallower and only contained one person’s remains (Additional File 1: Fig. S2). The arrangement of the remains from grave 12 clearly indicates the disrespectful nature of the burial. The bodies were positioned in unnatural positions, as if having been thrown in, with legs curled up. It seems likely that the person buried in grave 13 had to first bury six people in grave 12 and later was also murdered and buried in the neighbouring grave. Next to almost all the remains, some pieces of fabrics, leather and other small items, such as buttons, knives, spoons, belt buckles and leather shoes, were found.

Another skeletal grave (grave 14) was discovered at a distance of about 120 m to the south-east from graves 12 and 13, in the area of mass grave 7. A person in grave 14 was also buried in clothes, of which some buttons were preserved, and additionally, parts of shoes were found. This burial differed from the rest in the way the body was placed. The individual from grave 14 seemed to have been arranged more carefully, in a straight position on the back, with the head pointing north and hands alongside the body (Additional File 1: Fig. S3).

Skeletal grave 16, with dimensions 1.60 × 1.10 and depth of approximately 0.80 m, was discovered in the autumn of 2013. It was located about 15 m north of grave 14. The remains of two people rested at the bottom of the pit, with their heads pointing to the east. The person buried next to the north wall was lying in an upright position, whereas the person at the south wall was lying on the left side of the body, with the legs slightly curled up (Additional File 1: Fig. S4). The latter person was clearly taller than the other and the grave seemed to be too small. A few artefacts were found near the remains, mostly personal items, which did, however, not reveal any details about the origins of the victims. An external examination of the bones of both individuals did not show any traces of the use of weapons, therefore, the cause of death of both persons remains unexplained.

### Anthropological and medico-legal assessments of the recovered human remains

Anthropological analyses were performed on the skeletal remains of ten individuals from four graves [12, 13, 14 and 16] . The list of collected data comprised the estimated age, stature and the biological sex of the exhumed individuals. Furthermore, the preservation states of the skeletons and bones thereof were evaluated, possible anomalies pathologically assessed and a trauma analysis was performed. Bones were studied by using generally accepted methods of skeletal analysis and bone description [26–29]. The age-at-death of individuals was estimated on basis of a comprehensive multivariate analysis of age-related changes in morphological features of bones and teeth. The list of physical characteristics included cranial suture obliteration, teeth abrasion, pubic symphysis morphology and auricular surface, sternal ends of the ribs, general skeletal ossification and signs of degenerative changes [26, 27, 30–33]. Estimation of the biological sex relied on generally accepted metric and descriptive pelvis and skull features, showing the sexual dimorphism to the greatest extent [27, 30]. Measurements of metric skeletal traits were performed in accordance with the so-called Martin technique [34]. Stature estimation of adult individuals was performed by regression analysis of length data, obtained from the long bone’s measurements [35, 36].

Table S1 (Additional File 1) summarises the results obtained by the anthropological examinations. The ten excavated skeletons were attributed to men, who were estimated to be between 20 and 60 years of age at the time of their death. The deceased were characterised by weakly arched, moderately high and short skulls, featuring rather narrow faces, big eye sockets and narrow, long noses. The individuals were medium or short in stature (Additional File 1: Table S1). Four skulls showed traces of injuries caused by a firearm (skeletons 2, 4, 5, 6) and skeleton number 8 displayed signs of injuries caused by a gunshot to the chest (Additional File 1: Table S1). All entry wounds had a diameter of 7–8 mm. The remaining five skeletons (I, II, 1, 3, 7) showed damages that may have resulted from gunshots or were otherwise caused by the bad preservation state. The localization of the entry wounds pointed to a systematic way of execution of the victims: shots (usually a single one) were aimed exclusively to the back of the head or near the neck. The gunshot injuries were characterised by an entry wound in the occipital bone, a gunshot channel running obliquely upwards and an exit wound in the frontal or orbital bone (in the median line of the body). This pattern indicated that the bullets were fired from a short weapon (it is more difficult to take such a shot from a shoulder arm) to a victim lying down or kneeling, with the head strongly inclined downwards. The lack of bullets in the skull cavities suggested the use of a weapon with high kinetic energy ammunition, which caused through-and-through injuries and extensive bone fractures, leading to secondary cracks on the frontal bone and partial fragmentation of the skulls (Additional File 1: Fig. S5).

### Analysis of artefacts revealed in the former extermination camp in Sobibór

Three of the artefacts found during the archaeological work were later subjected to ballistic analyses: two brass pistol shells and one iron-made pistol projectile (Additional File 1: Fig. S6-7).

Both shells were of calibre 7.65 mm and could be assigned to the Browning-system, which was typical for self-loading pistols. This system was commonly used in weapons like Walther PPK, Walther PP, Browning pistols or Mauser HSC. Some of these (e.g. Walther) handguns were standard equipment of German guards in concentration and death camps [1]. The analysis of the shells suggested that they were fired from two different pieces or even types of weapons.

Both pistol shells and the corroded pistol projectile could be assigned to the *Deutsche Waffen- und Munitionsfabriken Aktien-Gesellschaft* (Eng. German Weapons and Munitions public limited company), known as DWM. Shell number 1 carried the clearly visible stamped-in number “DWM 479A BB” (Additional File 1: Fig. S8), which is a catalogue number from DWM Berlin-Borsigwalde.

Altogether more than 100 artefacts were recovered during the fieldwork at the former extermination camp in Sobibór. Most of these included pieces of metal and fabric, which did not carry any specific information about the victims. However, three artefacts appeared to be personal belongings: a pocketknife (Additional File 1: Fig. S9), a leather shoe (Additional File 1: Fig. S10) and a metal spoon (not shown). Even though these objects did not suggest much about the identity of the victims, they could be rather classified as belongings of civilians.

### Bone material preparation

All samples addressed in this study were collected by the Department of Forensic Genetics at the Pomeranian Medical University in Szczecin, Poland. The genetic testing was commissioned by the Institute of National Remembrance in Lublin and was a part of a process of identifying the remains of victims of communist crimes committed in Poland, as originally assumed.

According to the procedures introduced by the Polish Genetic Database of Victims of Totalitarianisms [3] whole healthy molars and 2 × 2 cm fragments of femoral shafts were used for DNA extraction and testing in a first step. Specimens were first mechanically cleaned using a precision drill with sterile diamond grinding bits and then chemically treated by a 5-min soaking step in a 1% sodium hypochlorite solution. Subsequently, the samples were washed with distilled water and air-dried in a laminar flow chamber. Dried teeth and femur pieces were frozen in liquid nitrogen and then milled to a powder, using a 6870 Freezer/Mill® (Spex SamplePrep, Metuchen, NJ).

### DNA extraction

Two different protocols were used for DNA extraction. The first one was based on magnetic particles technology for which the PrepFiler® BTA Forensic DNA Extraction Kit by Thermo Fisher Scientific (TFS, Waltham, MA) was used. Fifty milligrams of bone or tooth powder were used for lysis with 220 μl BTA Lysis Buffer, 3 μl 1 M dithiothreitol and 7 μl proteinase K solution (20 mg/ml). Samples were lysed for 2 h in a thermal shaker, set at 1100 rpm and 56 °C. All further extraction steps complied with the manufacturer's instructions.

The second protocol relied on a phenol-chloroform method for DNA extraction [37]. Bone powder (1 g) was lysed overnight with 2.5 ml buffer (0.5% 0.5 M ethylenediaminetetraacetic acid, 0.5% Tween 20) and 85 μl proteinase K (20 mg/ml) at 56 °C. After lysis, the remaining tissue was pelleted by centrifugation (10,000 rpm for 2 min). The resulting supernatants were transferred into new sterile tubes and mixed with an equal volume of phenol to chloroform to isoamyl alcohol (25:24:1, pH 8.0). After phase separation by centrifugation, the upper, aqueous layer was recovered and subjected to two additional rounds of phenol treatment. The DNA extraction process was completed by two rounds of silica-matrix based purification by means of the QIAquick PCR Purification kit (Qiagen, Hilden, Germany) according to the vendor’s protocol. Finally, the DNA was eluted in 50 μl buffer EB (Qiagen).

### DNA quantification

The DNA extracts obtained for all specimens were quantified for their mtDNA content using an “in-house” real-time quantitative PCR method [38, 39]. The reactions were performed on a 7500 fast Real-Time PCR System and results were analysed with the 7500 Software v2.0 (both TFS) (Additional File 1: Table S2).

### Mitochondrial lineage marker analyses

According to the procedures used by the Polish Genetic Database of Victims of Totalitarianisms, the HV1 (16,024–16,365) and HV2 (73–340) segments of the human mtDNA control region were sequenced using the Sanger method. The following four primer pairs were used: L15997/H16236, L16190/H16401, L048/H285 and F155/H408. The 20 μl singleplex PCR assays comprised of 1x HotStarTaq Master Mix (Qiagen), 500 nM (each) forward and reverse primer and 3 μl total genomic DNA (samples of interest, positive controls) or H_2_O (no template controls). Thermal cycling on a Veriti 96-well PCR instrument (TFS) consisted of initial denaturation at 95 °C for 15 min, followed by 35 cycles of denaturation at 94 °C for 30 s, primer annealing at 50 °C for 30 s and primer extension at 72 °C for 1 min. The final extension step was prolonged by 10 min. For post-PCR removal of unincorporated dNTPs and primers, the ExoSAP-IT reagent (TFS) was used according to the manufacturer’s instructions. The 20 μl Sanger sequencing reactions were composed of 0.5x BigDye™ Terminator v3.1 Ready Reaction Cycle Sequencing mix, 1x BigDye™ Terminator 5X Sequencing Buffer (both TFS) and 160 nM forward or reverse amplification primers. Thermal cycling on a Veriti instrument comprised an initial hold at 96 °C for 1 min and 25 cycles of 95 °C/15 s, 50 °C/5 s and 60 °C/4 min. Sequenate purification was performed with the ExTerminator kit (A&A Biotechnology, Gdynia, Poland), for which the provided protocol was followed. Electrophoretic separation of the sequencing products was conducted on a 3130 Genetic Analyzer (TFS). The resulting electropherograms were analysed with the Sequencher DNA Sequence Analysis Software (v5.4.6, Gene Codes, Ann Arbor, MI). On basis of the HV1/HV2 mitotypes, the haplogroup affiliations of the samples of interest were estimated by utilising routines implemented in the EMPOP database [13].

Full mitogenomes were sequenced using the Precision ID mtDNA Whole Genome Panel (TFS). The assay included two overlapping primer pools (81 primer pairs each) that target the entire mitogenome. Library preparation was performed with 6 μl DNA (12 μl for both primer pools) input and the “full” method (each pool was amplified separately) [40], as recommended by the manufacturer. PCR amplification was performed on a GeneAmp PCR System 9700 (TFS), with the following thermal profile: 99 °C/2 min (99 °C/15 s; 60 °C/4 min) × 21; 10 °C/∞. After the amplification step, a partial digestion of the primers with FuPa enzyme (TFS) was performed and ligate barcode adapters (TFS) were added (using the same barcode for both pools). The libraries were purified with AMPure XP beads and, after elution, they were quantified using the Ion Library TaqMan Quantitation Kit (both TFS). The libraries were diluted to 30 pM and then pooled at equal volumes. The remaining steps of library preparation were performed in an automated way by using the Ion Chef Instrument (TFS) and following the manufacturer’s recommendations. Samples were loaded on a 530 Chip and sequencing was performed on an Ion S5 System (both TFS) [40]. Sequencing data were analysed with the Torrent Suite 5.2.1 software (TFS) and differences to the revised Cambridge Reference Sequence [41] were reported using the variantCaller (v5.2.1.38) plug-in. The sequences were also analysed with IGV (Integrative Genomics Viewer) (http://software.broadinstitute.org/software/igv/) [42] software. A total of 22 DNA extracts were analysed from the ten persons. All of them yielded sequence data covering the entire mitogenome. Haplogrouping was performed using EMPOP (https://empop.online; v4/R13; Additional File 1: Table S3).

### Statistical and phylogenetic analysis

In order to statistically evaluate the established mtDNA haplotypes, we queried them against a set of 945 Polish haplotypes (as this was the assumed nationality of the remains) available in the EMPOP database and against a set of 744 Ashkenazi haplotypes available in literature [4, 6]. The resulting probabilities were calculated using the augmented counting method (x + 1)/(n + 1) (Additional File 1: Table S5). The ranges of mitotypes were adjusted for both searches according to the available data. The matches found in the Polish population were not listed amongst the matches presented in Table S4 (Additional File 1) [43–48], because the queried Polish dataset does not cover HVS-III.

In addition to studying the geographical distribution of obtained haplotypes and their statistical evaluation, the phylogenetic relation between the mitogenomes of the remains and of two populations in question, namely Poles and modern AJ, was analysed. We used a dataset consisting of 540 Polish and 88 Jewish mitogenomes (Additional File 1: Table S6) and selected individuals corresponding to seven haplogroups detected in the remains (K, H3, HV, I, J, V and X), which resulted in a total of 140 mitogenomes. For each haplogroup, multiple sequence alignments were performed in MEGA X [49, 50] using MUSCLE and Neighbour Joining as clustering methods [51]. The evolutionary history was inferred by using the Maximum Likelihood method and Tamura 3-parameter model [52]. Initial trees for the heuristic searches were obtained automatically by applying Neighbour-Join and BioNJ algorithms to a matrix of pairwise distances estimated using the Tamura 3 parameter model. This was followed by selecting the topology with superior log likelihood value.

### Analysis of Y-chromosomal markers

We employed the Yfiler Plus PCR Amplification Kit (TFS) for multiplex Y-STR analysis. Thirty cycle PCR amplifications were performed on a Veriti thermal cycler following the protocol recommended by the manufacturer. Positive and negative controls were amplified in parallel to the samples of interest. Electrophoretic sizing of the PCR products was performed on a 3500 Genetic Analyzer, using a 36 cm capillary array, POP-4 polymer, GeneScan 600 LIZ™ as internal size standard and the GeneMapper® ID-X software for data analysis (all TFS). Y-STR haplotypes were obtained for all individuals with five of them being partial. The data was used for haplogroup estimation using the NevGen Y-DNA Haplogroup Predictor software (http://www.nevgen.org/).

Direct Sanger sequencing of singleplex PCR products was used to verify the Yfiler Plus haplotype based NevGen Y-SNP haplogroup predictions. For this purpose, seven Y-SNP loci (Additional File 1: Table S7) were chosen under consideration of relevant information found on the literature [17–23]. While markers M123 and P58 complied with the NevGen prediction for the most derived Y-SNP locus, the other five markers tested for actual haplogroup membership at a level higher up in the Y-chromosomal phylogeny than that suggested by the software. Amplicon lengths were adjusted to account for the high degradation state of the biological material and ranged between 101 and 241 bp (Additional File 1: Table S8).

The 20 μl amplification reactions comprised of 5 μg non-acetylated bovine serum albumin, 1x buffer II, 2 mM MgCl_2_, 200 μM each dNTP, 400 nM (each) forward and reverse primer (Additional File 1: Table S8), 2 units (P58) or 2.25 units (remaining markers) of AmpliTaq Gold DNA polymerase and 5 μl DNA extract (samples of interest, positive control: 100 pg) or H_2_O (no template controls). Thermal cycling was conducted in 96-well polypropylene plates on a GeneAmp PCR System 9700 instrument. The thermocycler protocol for P58 amplification consisted of initial denaturation at 95 °C for 10 min, followed by 10 touchdown cycles of 95 °C/15 s, 70 °C → 61 °C/30 s and 72 °C/45 s (annealing temperature decrement − 1 °C/cycle) and 35 cycles of 95 °C/15 s, 60 °C/30 s and 72 °C/45 s. The final extension step was prolonged by 10 min. For PCR amplification of the remaining six Y-SNP loci, we applied initial denaturation at 95 °C for 10 min, followed by 10 touchdown cycles of 95 °C/15 s, 70 °C → 60 °C/30 s and 72 °C/30 s (annealing temperature decrement − 1 °C/cycle) and 38 cycles of 95 °C/15 s, 60 °C/30 s and 72 °C/30 s. The final extension step was prolonged by 10 min. All further experimental steps followed the Sanger-type sequencing workflow outlined below for DYS388 and DYS426.

### Cohen Modal Haplotype

The extended Cohen Modal Haplotype (CMH) [18] comprised 12 Y-STR markers: DYS19, DYS388, DYS390, DYS391, DYS392, DYS393, DYS385a/b, DYS389I, DYS389II, DYS426 and DYS439. The Yfiler Plus kit amplified all but two of these loci—DYS388 and DYS426 were missing. Direct Sanger sequencing of DYS388 and DYS426 single-plex PCR products for four individuals, assigned to haplogroup J-P58 (S5, S7, S9, S10), was performed. The 20 μl amplification reactions comprised of 5 μg non-acetylated bovine serum albumin (Sigma-Aldrich, St. Louis, MO), 1x buffer II, 2 mM MgCl_2_, 200 μM each dNTP (all TFS), 400 nM (each) forward and reverse primer, 2.25 units AmpliTaq Gold DNA polymerase (TFS) and 5 μl DNA extract (samples of interest, positive control) or H_2_O (no template controls). Thermal cycling was conducted in 96-well polypropylene plates on a GeneAmp PCR System 9700 (TFS) instrument. The cycler protocol consisted of initial denaturation at 95 °C for 10 min, followed by 10 touchdown cycles of 95 °C/15 s, 66 °C → 57 °C/30 s and 72 °C/30 s (annealing temperature decrement − 1 °C/cycle), and 39 cycles of 95 °C/15 s, 56 °C/30 s and 72 °C/30 s. The final extension step was prolonged by 10 min. Post-PCR treatment of amplicons was performed with the ExoSAP-IT enzyme blend, as recommended by the vendor. The sequencing reagent mixes contained 2 μl BigDye™ Terminator v1.1 Ready Reaction Cycle Sequencing mix, 2 μl BigDye™ Terminator 5X Sequencing Buffer (all TFS), 160 nM forward or reverse amplification primer and water to 10 μl. Thermal cycling on a GeneAmp PCR System 9700 instrument comprised an initial hold at 96 °C for 1 min and 30 cycles of 95 °C/15 s, 50 °C/5 s and 60 °C/4 min. Sequenates were purified by centrifugation over Optima DTR 96-well plates, as recommended by the manufacturer (Edge BioSystems, Gaithersburg, MD). Electrophoretic separation of the cycle sequencing products was performed on a 3100 Genetic Analyzer (TFS), using a 36 cm capillary array and POP-6 as sieving matrix. Sequencing traces were analysed with the Sequencher software (Gene Codes, v5.1, build 10627).

## Supplementary Information

**Additional File 1: Fig. S1**. Localization of 22 graves discovered in Sobibór during the archaeological research. All were located within the area of *Lager III* / Camp III. Graves numbered 1–7 and 8/15 were mass graves, graves 9–11 and 17–20 were small cremation graves and graves 12–14, 16 (marked in blue) were unexpected skeletal graves. **Fig. S2.** Grave 12 (right) containing six burials and grave 13 (left) with only one person’s remains. **Fig. S3.** The individual from grave 14 was buried in a straight position on the back with the head pointing north and the hands alongside the body. **Fig. S4.** Grave 16 with the remains of two people. **Fig. S5.** Four skulls showed traces of injuries caused by firearm with entry wounds of 7-8 mm in diameter. **Fig. S6.** One of the two brass pistol shells of calibre 7.65 mm subjected to ballistic analyses and assigned to the Browning-system, which was typical for self-loading pistols e.g., Walther handguns (standard equipment for German guards in concentration and death camps). **Fig. S7.** Corroded iron-made pistol projectile. **Fig. S8.** Pistol shells were assigned to the *Deutsche Waffen- und Munitionsfabriken Aktien-Gesellschaft* (Eng. German Weapons and Munitions public limited company). The stamped-in number “DWM 479A BB” refers to a catalogue number from DWM Berlin-Borsigwalde. **Fig. S9.** One of a few preserved artefacts: a pocket knife. **Fig. S10.** One of a few preserved artefacts: a leather shoe. **Table S1.** Summary of the anthropological examinations. The ten excavated skeletons were attributed to men, who were estimated between 20 and 60 years at the time of death. Five of them showed clear gunshot traumas. **Table S2**. Summary of mtDNA-specific quantification results in all extracts submitted for sequencing (number of mtDNA genome equivalents per μl) [38, 39]. **Table S3.** Consensus mitogenome sequences observed in the ten remains and reported relative to the rCRS and corresponding haplogroup assignment. **Table S4**. Summary of mtDNA database queries for each individual remain (S1-S10). The mtDNA control region was searched in EMPOP, full mitogenome searches were performed in a vetted set of 27,737 mitogenomes from GenBank. **Table S5.** Summary of the remains’ haplotype searches in a Polish population sample (*n* = 945) and in a set of Ashkenazi Jews (*n* = 744). **Table S6.** GenBank numbers of mitogenomes used for phylogenetic analysis of Poles and Ashkenazi Jews [15, 16]. **Table S7**. Y-STR haplotypes and corresponding haplogroup estimations. **Table S8**. Summary of the Y-SNP-sequencing results [53–75].

**Additional file 2.** Review history

## Data Availability

The genetic raw data generated in the course of this study are not publicly available due to nature of the case (see the “Methods” section for details).
