# Supplementary material for: Genetic and phylogeographic evidence for Jewish Holocaust victims at the Sobibór death camp
Source: Genome Biol. 2021 Aug 6;22:200. doi: 10.1186/s13059-021-02420-0 (PMC8343952; doi:10.1186/s13059-021-02420-0)
Supplement: Supplementary file 1 — Additional File 1: Fig. S1. Localization of 22 graves discovered in Sobibór during the archaeological research. All were located within the area of Lager III / Camp III. Graves numbered 1–7 and 8/15 were mass graves, graves 9–11 and 17–20 were small cremation graves and graves 12–14, 16 (marked in blue) were unexpected skeletal graves. Fig. S2. Grave 12 (right) containing six burials and grave 13 (left) with only one person’s remains. Fig. S3. The individual from grave 14 was buried in a straight position on the back with the head pointing north and the hands alongside the body. Fig. S4. Grave 16 with the remains of two people. Fig. S5. Four skulls showed traces of injuries caused by firearm with entry wounds of 7-8 mm in diameter. Fig. S6. One of the two brass pistol shells of calibre 7.65 mm subjected to ballistic analyses and assigned to the Browning-system, which was typical for self-loading pistols e.g., Walther handguns (standard equipment for German guards in concentration and death camps). Fig. S7. Corroded iron-made pistol projectile. Fig. S8. Pistol shells were assigned to the Deutsche Waffen- und Munitionsfabriken Aktien-Gesellschaft (Eng. German Weapons and Munitions public limited company). The stamped-in number “DWM 479A BB” refers to a catalogue number from DWM Berlin-Borsigwalde. Fig. S9. One of a few preserved artefacts: a pocket knife. Fig. S10. One of a few preserved artefacts: a leather shoe. Table S1. Summary of the anthropological examinations. The ten excavated skeletons were attributed to men, who were estimated between 20 and 60 years at the time of death. Five of them showed clear gunshot traumas. Table S2. Summary of mtDNA-specific quantification results in all extracts submitted for sequencing (number of mtDNA genome equivalents per μl) [38, 39]. Table S3. Consensus mitogenome sequences observed in the ten remains and reported relative to the rCRS and corresponding haplogroup assignment. Table S4. Summary of mtDNA database queries for eac [file 13059_2021_2420_MOESM1_ESM.docx]

Additional file 1

Genetic and phylogeographic evidence for Jewish Holocaust victims at the Sobibór death camp

Marta Diepenbroek, Christina Amory, Harald Niederstätter, Bettina Zimmermann, Maria Szargut, Grażyna Zielińska, Arne Dür, Iwona Teul, Wojciech Mazurek, Krzysztof Persak, Andrzej Ossowski, Walther Parson

Correspondence to: andrzej.ossowski@pum.edu.pl; walther.parson@i-med.ac.at

Supplementary Text

The Sobibór death camp – historical background

The euphemism “final solution of the Jewish problem” (German: *die Endlösung der Judenfrage*) used in the parlance of the Nazi government of the Third Reich denoted the idea of exterminating Jews of Europe during the Second World War. The persecution of Jews in Germany begun immediately after Hitler’s rise to power in 1933. Persecution went through the stage of the policy of forced emigration and expulsion, later transforming into a mass murder of nearly six million Jews during the war (*53*).

The German invasion of Poland in 1939 and the beginning of the Second World War facilitated the radicalization of the Nazi anti-Jewish policy. On the newly occupied territories, Jews were concentrated in ghettos, where dozens of thousands died of hunger and diseases, which was a form of indirect extermination. At that stage, the Nazis considered various versions of “territorial solutions”, involving the deportation of Jews to a “reservation” in the area of Lublin, Poland, or even to Madagascar, where they were supposed to perish (*54*). The Nazis were determined to turn their realm free of Jews (*judenrein*), but still did not have a precise idea how to do it in practice.

Germany started the direct mass extermination of Jews after its attack on the USSR in the summer of 1941. Although only Communists and Jewish men were to be shot according to the initial orders issued to *Einsatzgruppen* (Eng. ‘Task Forces’), *Schutzstaffeln* (SS) (Eng. ‘Protection Squadrons’) and other formations (paramilitary units of Nazi Germany), very soon executions encompassed also Jewish women and children. At the Eastern Front, the Germans also started experimenting with various methods of mass killing (*55*). Members of the Nazi elite equated the war against the Communist Soviet Union with the war against Jews. This mental shift, coinciding with murderous initiatives of zealous SS commanders, brought the idea of killing all Jews into the sphere of realistic options. The results of the Holocaust research of the recent two decades suggest that there was no single general decision about the extermination of Jews taken at a certain moment. It was rather a dynamic process, and the idea of exterminating Jews ripened in the late fall of 1941 (*56*).

Individual initiatives of local Nazi governors, who anticipated Adolf Hitler’s and Heinrich Himmler’s (*Reichsführer*-SS, national leader of the SS) intentions, were also of crucial importance. Already in December 1941, Arthur Greiser, Gauleiter of German-occupied western Poland (Warthegau), launched the first instant extermination centre in Chełmno (Kulmhof) near Łódź. He employed the experience and personnel members of the euthanasia operation of the disabled and mentally ill patients, known under the code-name T4, which had been soon halted in Germany due to protests of the public opinion (*57*).

The infamous conference in Wannsee near Berlin, which the head of the Reich’s Main Security Office Reinhardt Heydrich convened on 20 January 1942, sealed the earlier decisions on exterminating the European Jews. Representatives of the Nazi Party, state administration and the SS who attended the conference, discussed the coordination of their actions and responsibilities (*58*). Josef Bühler, who appeared in Wannsee on behalf of the General Governor of German-occupied central Poland, Hans Frank, insisted on beginning the “Final Solution” in the General Government as quickly as possible. He also hinted at the necessity to discretely initiate “certain preparatory activities” (*59*).

Bühler might have alluded to the extermination camp in Bełżec, which was already under construction on the initiative of the SS and Police commander in the Lublin District of the General Government, Odilo Globocnik. The latter, a trusted and ambitious Himmler’s aide, played a pivotal role in the extermination of Polish Jewry. He oversaw an operation, which was code-named “Reinhardt” after Heydrich’s death. Its only objective was to kill Jews, living in the General Government and later also in the neighbouring Białystok District of East Prussia. Among important Globocnik’s aides were Hermann Höffle – the Chief of Staff of the Operation Reinhardt, Christian Wirth – the General Inspector of the extermination centres and Richard Thomalla, who designed and supervised the construction of the death camps. A major group of SS-men, who ran the death camps under Globocnik, were veterans of Operation T4 (*60*).

Operation Reinhardt begun on the night of 16-17 March 1942 when the Nazis started the liquidation of the ghetto in Lublin by deporting its Jews to the extermination camp in Bełżec. In the following months, Globocnik established two more extermination camps in Sobibór (May 1942) and Treblinka (July 1942). Jews from the ghettos in German-occupied Poland were deported there and instantly killed in gas chambers. Among the victims of Operation Reinhardt were also Jews from Germany, Austria, Czech Lands and Slovakia, who had been previously deported to emptied ghettos in the Lublin District. Groups of Jews from other countries – the Netherlands, France, Greece, Macedonia and Belarus – were also sent directly to Operation Reinhardt death camps (*61*).

Auschwitz-Birkenau and Majdanek were both concentration camps and extermination camps. Bełżec, Sobibór and Treblinka, on the other hand, had never been designed to detain prisoners, except for relatively small groups who were necessary to ensure the functioning of the camps. They served only one purpose: the industrial scale mass murder. The estimated numbers of victims amounted up to 450,000 in Bełżec (*62*), up to 180,000 in Sobibór (*63*) and around 800,000 in Treblinka (*64*). During Operation Reinhardt, Polish Jews did not only die in extermination camps. They were also murdered in the course of “actions” of liquidated ghettos and killed during mass executions. The final part of Operation Reinhardt was the action “*Erntefest*” (Harvest Festival) on 3–4 November 1943, when the Germans shot 42,000 Jewish prisoners at Majdanek and two other labour camps in the Lublin District. The overall death toll of Operation Reinhardt is estimated at nearly two million people (*65*). After the extermination facilities in Bełżec, Sobibór and Treblinka fulfilled their deadly objective, the Germans demolished them and scrupulously obliterated their traces.

The Sobibór death camp was the second instant extermination centre built in the Lublin District within the framework of Operation Reinhardt. Initially, it was probably designed as an establishment subsidiary to the Bełżec death camp. It was located in a sparsely populated area among the woods, 4 kilometres away from the village Sobibór, at the eastern border of the Lublin District. Such a location facilitated securing secrecy around the new “death factory”, while its position on the Lublin-Chełm-Włodawa railway track made it possible to easily import new Jewish victims. However, these precautions turned out to be only moderately effective and reports about a new death camp in Sobibór appeared in the Polish clandestine press already within two months after (*66*).

The design of the Sobibór extermination centre followed the pattern of Bełżec. Also here, the construction was supervised by Richard Thomalla. The existing buildings, belonging to the forestry inspectorate, were incorporated into the camp and served as the commander’s headquarters and barracks for the German personnel (the so-called *Vorlager*). Franz Stangl was appointed first commander of SS-*Sonderkommando* Sobibór, which was the camp’s official name. After Stangl had been transferred to Treblinka, Franz Reichleitner replaced him and maintained the position of commander until the liquidation of the camp in December 1943. The Sobibór crew was composed of 20–30 SS-men (ethnic Germans and Austrians) and about 130 watchmen, recruited from among Soviet prisoners of war, predominantly of Ukrainian descent, who were instructed in the special training camp in Trawniki, established by Globocnik (*67*).

The first transports of victims arrived in Sobibór at the turn of April and May 1942. Initially, Jews from the northern part of the Lublin District were exterminated there. However, already by the end of May, Jews from Germany, Austria and the Protectorate of Bohemia and Moravia, earlier deported to transit ghettos in the Lublin area, were sent to Sobibór. In the later period also Jews from Slovakia, France, the Netherlands and Belarus were killed in the camp. The death toll of Sobibór is estimated at between 170,000 and 180,000, of which Jews from outside pre-war Poland were around 45% with Jews from the Netherlands and Slovakia as the largest groups – 34,000 and 24,000 respectively. In Sobibór, the Germans killed also small groups of Roma (*63*).

All our knowledge about Sobibór is based on depositions of German and Ukrainian members of the camp crew, which were made during their post-war trials and testimonies of the few prisoners who participated in the uprising and mass escape in 1943 and managed to survive the war. No archival documentation of the camp has been preserved. A rare exception is a ciphered telegram from Hermann Höffle to Adolf Eichmann, reporting that 101,370 Jews were deported to Sobibór by the end of 1942 (*68*). A most recent discovery is a collection of photographs documenting everyday life of the German Sobibór crew taken by the camp’s deputy commander Johann Niemann (*69*).

The camp was surrounded with a barbed wire fence with several watch towers and a strip of minefield. The fence was interwoven with pine and fir tree branches in order to prevent strangers from seeing what was happening inside. Similar fences separated particular parts of the camp: the *Vorlager*, *Lager I* with living quarters and workshops for prisoners, *Lager II*, which was a section designated for receiving new victims, as well as sorting and storing their belongings and finally *Lager III* – the most strictly isolated death zone with gas chambers and mass graves (*70*).

The killing procedure was well thought out and organized in such a way that the victims would not realise their fate for as long as possible and would not put up any resistance. A railway track from the Sobibór station was extended, so that deportation trains could be brought directly into the camp. Wooden buildings at the ramp were guised to resemble a railway station. A group of 25 prisoners, forming the *Bahnhofkommando*, were responsible for unloading the transport. An SS officer informed the newcomers that they had arrived in a transit camp and that they would be further sent to forced labour “to the East”. They were ordered to leave their belongings behind, hand over all valuables, undress and prepare for a disinfecting bath. Men and women were separated, and the latter had their hair cut before being forced to the gas chamber. The elderly and the sick, who were unable to undergo this procedure, were taken by a narrow-gauge railway directly to the vicinity of mass graves and shot there. The same railway was used to transport corpses of those who died in the railway cars during the journey to Sobibór (*67,70*).

A narrow path, 250 metres long and fenced with barbed wire, connected *Lager II* with *Lager III*. The Nazis herded the naked victims through this passage, which they mockingly called the “road to heaven” (*Himmelfahrtstrasse*), directly to the gas chamber. To sustain the lie about the disinfection, the gas chamber was disguised as a bath with shower heads below the ceiling. Its three compartments could each accommodate 500 to 600 people and were connected by pipes with a petrol engine, producing exhaust gas that contained toxic carbon monoxide. The victims died within 20–30 minutes. Later, a group of 50 Jewish prisoners called *Sonderkommando* removed the corpses and dragged them to enormous mass graves (*67,70*). This terrible work, incomparable with anything else, was extremely exhausting, both physically and psychologically. As a result, prisoners belonging to the *Sonderkommando* were executed after a short time and replaced by others. Not a single member of the *Sonderkommando* from Sobibór survived the war. Since *Lager III* was strictly isolated from the rest of the camp, prisoners from the *Sonderkommando* did not participate in the resistance conspiracy or the revolt and thus had no possibility to escape.

In the summer of 1942, when new transports were halted due to maintenance works at the railway line leading to Sobibór, the Germans expanded the gas chambers and doubled their capacity. The killing process was resumed in October 1942. At that time, the Nazis begun liquidating the mass grave by exhuming the corpses and burning them. An excavator was used to retrieve the remains and to place them in a pit, where they were overpoured with kerosene and incinerated. This activity was a part of a wider operation, known as Operation 1005, which was ordered by Himmler to obliterate traces of Nazi mass crimes. Since then, bodies of people killed in the Sobibór gas chamber were immediately burned up at huge grids made of iron rails (*67*).

Only prisoners who were selected by the Nazis from incoming transports for employment at various kinds of work, which was necessary for the functioning of the camp, had a possibility to survive in Sobibór for longer. At some rare occasions, small groups of newcomers were also sent to other labour camps outside Sobibór. On average the number of prisoners working in Sobibór at one particular time amounted to 600–700, including a group of women. Their assignments included sorting the possessions of the murdered victims, work in shoemakers’, tailors’, carpenters’ and leatherworkers’ workshops and serving the SS officers and the guards. Members of the *Sonderkommando* emptied the gas chambers and disposed of bodies of the murdered victims. The Nazis conducted frequent selections among working prisoners (*Arbeitsjude*), and the executed persons were replaced by those who arrived in new transports.

The prisoners in Sobibór were fully aware that their fate was sealed. Regardless of all difficulties and constant supervision by the guards they undertook attempts at escape. Archaeological excavations in Sobibór revealed remnants of a tunnel the prisoners of the *Sonderkommando* had dug, in order to escape from behind the fence. In April 1943, their plan was prematurely exposed and the Nazis executed all members of that *Sonderkommando*. In July 1943, prisoners of the *Waldkommando*, who worked in the forest outside the camp, cutting firewood and tree branches used for masking the fence, ventured a partly successful escape. They attacked their guards with axes and some of them succeeded to flee. The remaining ones were executed in front of the rest of the prisoners, some of whom were also randomly selected to be shot as a measure of collective responsibility (*67,70*).

In the summer of 1943 new transports with Jews were coming to Sobibór infrequently, and the working prisoners felt that their end was approaching. At the same time some newcomers brought the news about the Jewish uprising in Warsaw, which was an inspiration to resist. A conspiratorial group arose among the prisoners, led by a Polish Jew, Leon Felhendler. However, the underground organisation lacked members with military experience. The arrival of a transport of Jewish Red Army prisoners of war from Minsk with lieutenant Alexander Pechersky on 23 September 1943 reversed this situation. The conspirators recruited Pechersky and placed him in command of a planned revolt. Leon Felhendler became his deputy (*71*).

On 14 October 1943, in accordance to a devised plan, the conspirators lured SS officers into workshops and storehouses on various pretexts. Once inside, the prisoners attacked them with knives and axes and seized their weapons. They also cut telephone wires. However, the Nazis coincidentally discovered the body of one of the SS men, which spurred the insurgents to accelerate their action. They called prisoners for a roll call, and Pechersky led them to storm the gate and the fence of the camp. Dozens fell under the machine gun fire from the watchtowers and on the minefield surrounding Sobibór. However, about 300 runaways managed to reach the forest. Many of them were subsequently killed or apprehended, either by the SS during a manhunt, or they perished while in hiding. The author of the Sobibór camp monograph, Marek Bem, identified 61 prisoners who survived the war. Ten SS officers and several Ukrainian guards were killed during the revolt. In revenge, the Nazis murdered nearly all prisoners who did not decide to join the revolt, or were not able to escape, as was the case for the strictly isolated *Sonderkommando* (*72,73*). The Sobibór revolt was a direct reason for the Nazis’ decision to conduct action “*Erntefest*” and kill all Jews still remaining in the General Government (*74*).

After the uprising, the Nazis decided to liquidate the Sobibór camp, which was the longest functioning extermination centre of Operation Reinhardt after Bełżec had been closed in December 1942 and Treblinka in the wake of the 2 August 1943 prisoners’ revolt. By the end of October 1943, a group of around 200 Jewish prisoners from Treblinka were brought to Sobibór to perform the demolition work. After they completed their job, they were executed and their bodies incinerated. The last group of 75 Jewish prisoners was assigned to obliterate all traces of the death factory – cover the cremation pits, level the ground and plant trees on the site. Having completed this task, they were killed by a shot in the back of their heads in December 1943 (*75*).


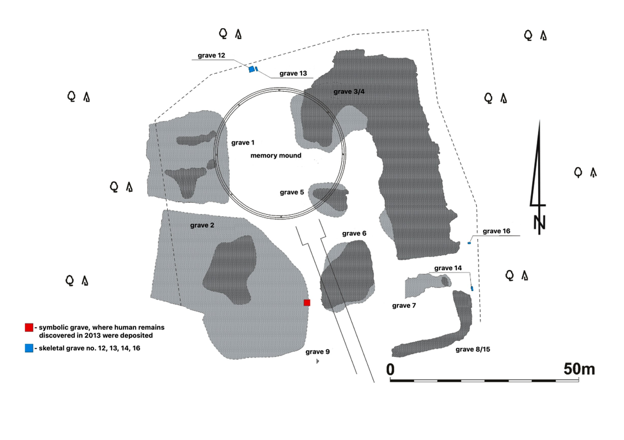


Fig. S1

Localization of 22 graves discovered in Sobibór during the archaeological research. All were located within the area of *Lager III* / Camp III. Graves numbered 1–7 and 8/15 were mass graves, graves 9–11 and 17–20 were small cremation graves and graves 12–14, 16 (marked in blue) were unexpected skeletal graves.


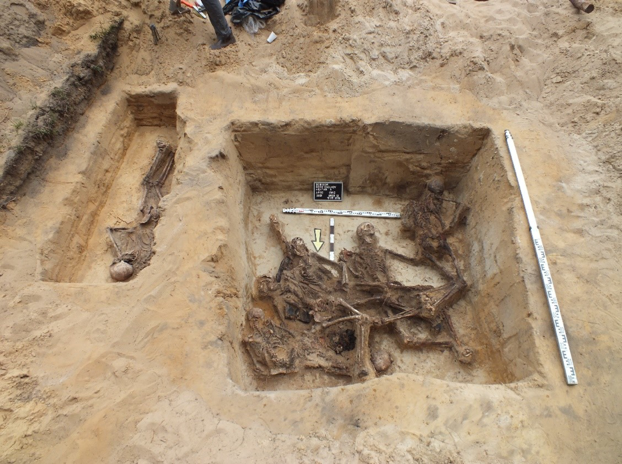


Fig. S2

Grave 12 (right) containing six burials and grave 13 (left) with only one person’s remains.


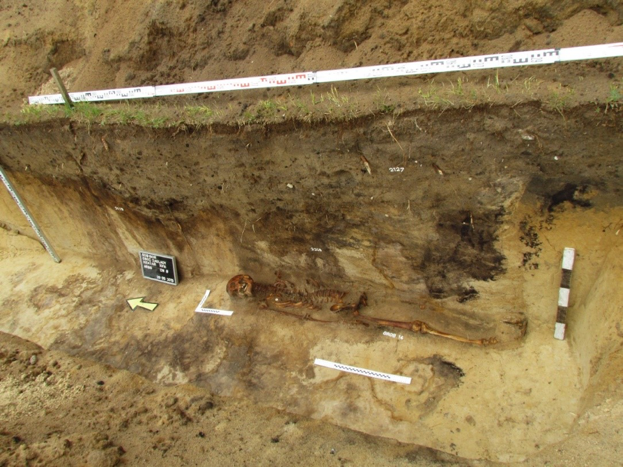


Fig. S3

The individual from grave 14 was buried in a straight position on the back with the head pointing north and the hands alongside the body.


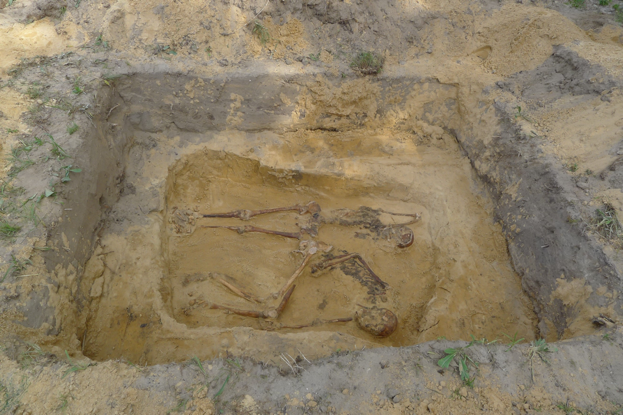


Fig. S4

Grave 16 with the remains of two people.


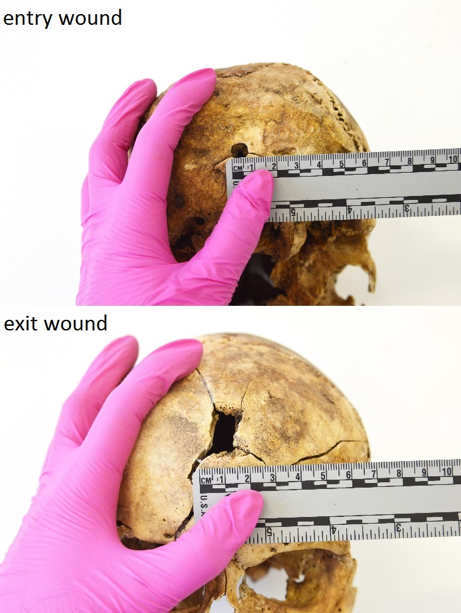


Fig. S5

Four skulls showed traces of injuries caused by firearm with entry wounds of 7-8 mm in diameter.


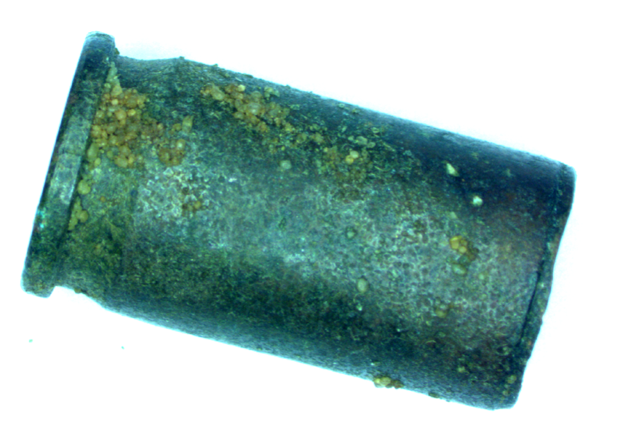


Fig. S6

One of the two brass pistol shells of calibre 7.65 mm subjected to ballistic analyses and assigned to the Browning-system, which was typical for self-loading pistols e.g., Walther handguns (standard equipment for German guards in concentration and death camps).


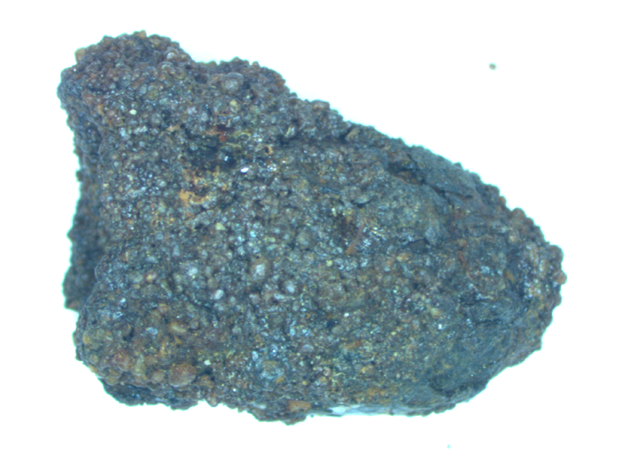


Fig. S7

Corroded iron-made pistol projectile.


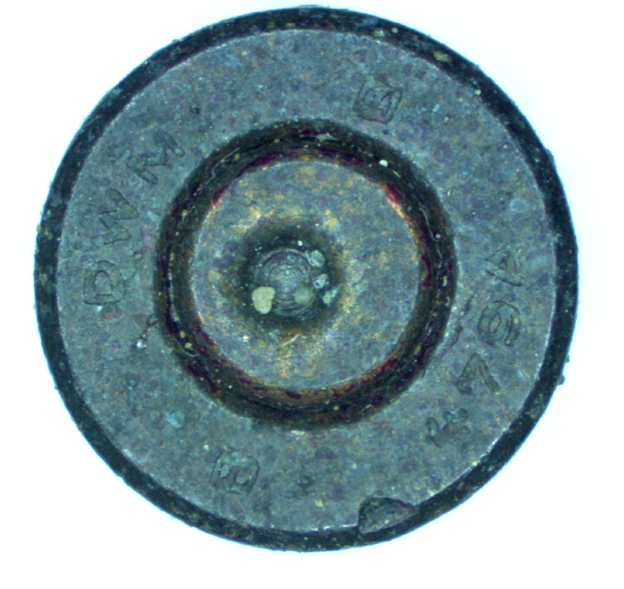


Fig. S8

Pistol shells were assigned to the *Deutsche Waffen- und Munitionsfabriken Aktien-Gesellschaft* (Eng. German Weapons and Munitions public limited company). The stamped-in number “DWM 479A BB” refers to a catalogue number from DWM Berlin-Borsigwalde.


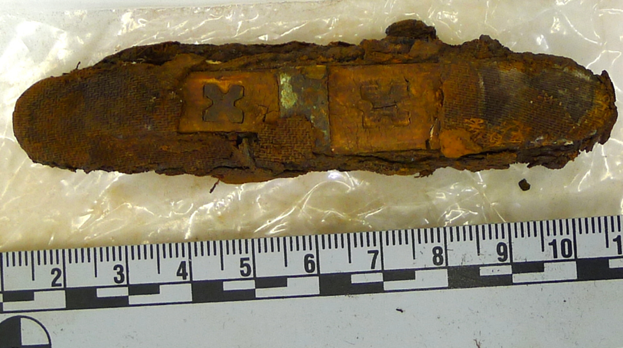


Fig. S9

One of a few preserved artefacts: a pocket knife.


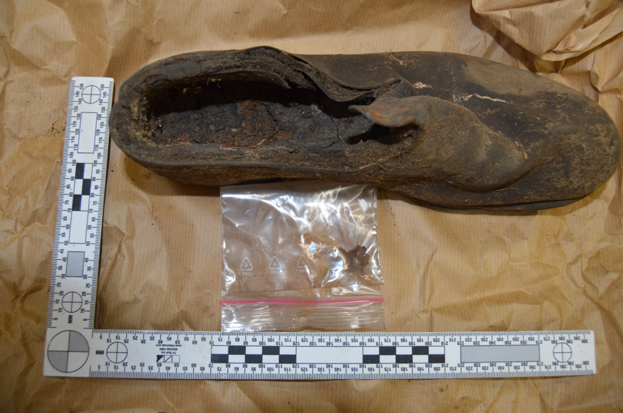


Fig. S10

One of a few preserved artefacts: a leather shoe.

| **Sample**  **no** | **Grave no** | **Skeleton no** | **Preservation of skeletal remains**  **(%) / state** | **Sex** | **Estimated age at death (years)** | **Estimated body height (cm)** | **Comments** |
| --- | --- | --- | --- | --- | --- | --- | --- |
| S1 | 16 | I | 75 / moderate | Male | 45–50 | 160–164 |  |
| S2 | 16 | II | 80 / good | Male | 25–30 | 157–159 |  |
| S3 | 12 | 1 | 80 / good | Male | 50–60 | 165–170 |  |
| S4 | 12 | 2 | 70 / moderate | Male | 20 | 158–161 | Perimortem trauma on occipital bone and on orbital bone, indicating 2 shots |
| S5 | 12 | 3 | 75 / moderate | Male | 50–60 | 161–165 |  |
| S6 | 12 | 4 | 65 / moderate | Male | 40–50 | 162–166 | Gunshot trauma on occipital and parietal bones |
| S7 | 12 | 5 | 80 / good | Male | 40 | 163–167 | Gunshot trauma on occipital and frontal bones |
| S8 | 12 | 6 | 85 / good | Male | 50 | 157–160 | Gunshot trauma on occipital and frontal bones |
| S9 | 13 | 7 | 75 / moderate | Male | 30–35 | 160–163 |  |
| S10 | 14 | 8 | 80 / good | Male | 25–30 | 155–157 | Gunshot trauma on sternum |

Table S1: Summary of the anthropological examinations. The ten excavated skeletons were attributed to men, who were estimated between 20 and 60 years at the time of death. Five of them showed clear gunshot traumas.

| Sample Name | mtGE/µL |  |
| --- | --- | --- |
|  |  |  |
| S1_1 | 757.2 |  |
|  |  |  |
| S1_2 | 512.9 |  |
|  |  |  |
| S2_1 | 583.7 |  |
|  |  |  |
| S2_2 | 561.8 |  |
|  |  |  |
| S3_1 | 1644.5 |  |
|  |  |  |
| S3_2 | 3617.5 |  |
|  |  |  |
| S3_3 | 387.2 |  |
|  |  |  |
| S4_1 | 1647.3 |  |
|  |  |  |
| S4_2 | 1574.8 |  |
|  |  |  |
| S5_1 | 417.0 |  |
|  |  |  |
| S5_2 | 70.0 |  |
|  |  |  |
| S6_1 | 137.6 |  |
|  |  |  |
| S6_2 | 1033.2 |  |
|  |  |  |
| S7_1 | 117.6 |  |
|  |  |  |
| S7_2 | 2739.5 |  |
|  |  |  |
| S8_1 | 1031.0 |  |
|  |  |  |
| S8_2 | 3967.8 |  |
|  |  |  |
| S9_1 | 809.6 |  |
|  |  |  |
| S9_2 | 1968.2 |  |
|  |  |  |
| S9_3 | 1336.1 |  |
|  |  |  |
| S10_1 | 1443.5 |  |
|  |  |  |
| S10_2 | 69.9 |  |
|  |  |  |
|  |  |  |

**Table S2:** Summary of mtDNA-specific quantification results in all extracts submitted for sequencing (number of mtDNA genome equivalents per µl) (*38,39*).

| **Individual** | **Haplotype** | **Haplogroup**  **(EMPOP; V4/R13)** |
| --- | --- | --- |
| S1 | 72C 89C 93G 195C 263G 315.1C 750G 930A 1438G 2706G 4580A 4769G 7028T 7444A 8860G 11899C 12753G 15326G 15904T 16153A 16298C | V7a |
| S2 | 146C 263G 315.1C 750G 1438G 4769G 6776C 8245G 8860G 9007G 15326G 15885T 16222T 16299G 16519C | H3p |
| S3 | 73G 114T 263G 315.1C 497T 750G 1189C 1438G 1811G 2706G 3480G 4769G 7028T 8860G 9055A 9698C 10398G 10550G 10978G 11299C 11467G 11470G 11719A 11914A 12308G 12372A 12954C 14167T 14766T 14798C 15326G 15924G 16093C 16223T 16224C 16234T 16311C 16519C | K1a1b1a |
| S4 | 73G 146C 152C 263G 315.1C 512C 709A 750G 1438G 1811G 2706G 3480G 4561C 4769G 7028T 8697A 8860G 9055A 9254G 9698C 9716C 10550G 11299C 11348T 11467G 11719A 11914A 12308G 12372A 14167T 14766T 14798C 15326G 16224C 16311C 16519C | K2a2a1 |
| S5 | 73G 114T 263G 315.1C 497T 750G 1189C 1438G 1811G 2706G 3480G 4769G 7028T 8860G 9055A 9698C 10398G 10550G 10978G 11299C 11467G 11470G 11719A 11914A 12308G 12372A 12954C 14167T 14766T 14798C 15326G 15924G 16223T 16224C 16234T 16311C 16519C | K1a1b1a |
| S6 | 152C 263G 315.1C 750G 1438G 2706G 3547G 4769G 6023A 7028T 8014T 8860G 12696C 15218G 15326G 16067T 16183M 16189Y 16519C | HV1b2 |
| S7 | 73G 146C 153G 195C 198T 204Y 225A 226C 263G 315.1C 750G 1438G 1719A 2706R 4216C 4769G 6221C 6371T 7028T 8393T 8860G 10634G 11719A 12705T 13708A 13966G 14110C 14470C 14766T 15326G 15927A 16183C 16189C 16223T 16278T 16519C | X2b7 |
| S8 | 73G 185A 195C 228A 263G 295Y 315.1C 462T 489C 750G 1438G 2706G 3010A 4216C 4491A 4769G 7028T 8860G 8940T 9120G 10398G 11251G 11719A 12612G 13708A 14766T 14798C 15326G 15452A 16069T 16126C 16261Y 16274R 16355T | J1c14 |
| S9 | 73G 146C 152C 263G 315.1C 512C 709A 750G 1438G 1811G 2706G 3480G 4561C 4769G 7028T 8697A 8860G 9055A 9254G 9698C 9716C 10550G 11299C 11348T 11467G 11719A 11914A 12308G 12372A 14167T 14766T 14798C 15326G 16224C 16311C 16519C | K2a2a1 |
| S10 | 73G 199C 204C 250C 263G 315.1C 455.1T 750G 1438G 1719A 2706G 4529T 4769G 6267A 6359G 6734A 7028T 8251A 8573A 8860G 9386C 9966A 10034C 10238C 10398G 11719A 12501A 12705T 13780G 14766T 15043A 15326G 15924G 16129A 16223T 16264T 16270T 16311C 16319A 16362C 16391A 16519C | I1c1a |

Table S3: Consensus mitogenome sequences observed in the ten remains and reported relative to the rCRS and corresponding haplogroup assignment.

| **Individual** | **Haplogroup** | **Control region matches**  **(EMPOP)** | | **Whole genome matches**  **(EMPOP+GenBank)** | |
| --- | --- | --- | --- | --- | --- |
|  |  | **Population**  **(number of matches)** | **Reference** | **Population**  **(number of matches)** | **Reference** |
| **S1** | **V7a** | AJ (4) | (*4*) | No matches | |
|  |  | USA (1) | (*13,14*) |  |  |
| **S2** | **H3p** | AJ (1) | (*4*) | AJ (1) | JQ704133 |
|  |  |  |  | USA (1) | (*41*) |
| **S3** | **K1a1b1a** | No matches | | AJ (1) | DQ301813 |
| **S4** | **K2a2a1** | AJ (14) | (*4*) | AJ (6) | KC757125; DQ301812; DQ301804; KX711887; HM347598; JQ705090 |
|  |  | USA (3) | (*13,14*) | Ukraine (1) | HQ914446 |
|  |  | Argentina (1) | (*13,14*) | Unknown (1) | EU327986 |
| **S5** | **K1a1b1a** | AJ (17) | (*4*) | AJ (7) | DQ301802; FJ228404; GU722599; GU723693; KF435080; KC914580; JQ702945 |
|  |  | USA (8) | (*13,14*) | USA (1) | (*42*) |
|  |  | Spain (4) | (*37*) | Germany (1) | JQ704654 |
| **S6** | **HV1b2** | AJ (1) | (*4*) | AJ (4) | EF421157; FJ210876; KC020246; JQ228861 |
|  |  | USA (2) | (*13,14*) | Poland (1) | KY782165 |
|  |  | Argentina (1) | (*38*) | Unknown (1) | DQ856316 |
| **S7** | **X2b7** | Basque (2) | (*39*) | Basque (2) | (*39*) |
|  |  |  |  | AJ (1) | JQ705082 |
| **S8** | **J1c14** | No matches | | AJ (1) | JQ705811 |
|  |  |  |  | Germany (1) | JQ705424 |
|  |  |  |  | Austria (1) | JQ702176 |
| **S9** | **K2a2a1** | AJ (14) | (*4*) | AJ (6) | KC757125; DQ301812; DQ301804; KX711887; HM347598; JQ705090 |
|  |  | USA (3) | (*13,14*) | Ukraine (1) | HQ914446 |
|  |  | Argentina (1) | (*13,14*) | Unknown (1) | EU327986 |
| **S10** | **I1c1a** | AJ (1) | (*4*) | AJ (1) | JQ705364 |
|  |  | USA (1) | (*13,14*) |  |  |
|  |  | Iraq (1) | (*40*) | USA (1) | (*42*) |
|  |  | Spain (1) | (*37*) |  |  |

**Table S4:** Summary of mtDNA database queries for each individual remain (S1-S10). The mtDNA Control Region was searched in EMPOP, full mitogenome searches were performed in a vetted set of 27,737 mitogenomes from GenBank.

| **Individual** | **Matches among Poles (n=945) (*13,14*)**  **Range=16024-16400** | **Probability**  **(x+1)/(n+1)** | **Matches among Ashkenazi (n=744) (*4,6*)**  **Range=16024-300** | **Probability**  **(x+1)/(n+1)** |
| --- | --- | --- | --- | --- |
| **S1** | 0 | 1,06e-3 | 12 | 1,74e-2 |
| **S2** | 0 | 1,06e-3 | 4 | 6,71e-3 |
| **S3** | 0 | 1,06e-3 | 3 | 5,37e-3 |
| **S4** | 7 | 8,46e-3 | 36 | 4,97e-2 |
| **S5** | 1 | 2,11e-3 | 63 | 8,59e-2 |
| **S6** | 0 | 1,06e-3 | 20 | 2,82e-2 |
| **S7** | 1 | 2,11e-3 | 2 | 4,03e-3 |
| **S8** | 0 | 1,06e-3 | 4 | 6,71e-3 |
| **S9** | 7 | 8,46e-3 | 36 | 4,97e-2 |
| **S10** | 0 | 1,06e-3 | 6 | 9,40e-3 |

**Table S5:** Summary of the remains’ haplotype searches in a Polish population sample (n=945) and in a set of Ashkenazi Jews (n=744).

| **GenBank number** | **Source** | **GenBank number** | **Source** | **GenBank number** | **Source** |
| --- | --- | --- | --- | --- | --- |
| DQ377992.1 | AJ (Belarus) | JQ704150.1 | AJ | MG952821.1 | Poland |
| EF396958.1 | AJ (Lithuania) | JQ704370.1 | AJ | MG952822.1 | Poland |
| EF419890.1 | AJ (Latvia) | JQ704427.1 | AJ | MG952823.1 | Poland |
| EF421157.1 | AJ (Lithuania) | JQ704870.1 | AJ | MG952824.1 | Poland |
| EF556150.1 | AJ | JQ704894.1 | AJ | MG952825.1 | Poland |
| EF556184.1 | AJ | JQ705236.1 | AJ | MG952826.1 | Poland |
| EF556189.1 | AJ | JQ705703.1 | AJ | MG952827.1 | Poland |
| EU148452.1 | AJ (Lithuania) | JQ898579.1 | AJ | MG952828.1 | Poland |
| EU148486.1 | AJ (Belarus) | JX025553.1 | AJ (Belarus) | MG952829.1 | Poland |
| EU262984.1 | AJ (Belarus) | KC020246.1 | AJ | MG952830.1 | Poland |
| EU558385.1 | AJ (Poland) | KC914580.1 | AJ (USA) | MG952831.1 | Poland |
| FJ228404.1 | AJ (Romania) | KF564293.1 | AJ (Czech) | MG952832.1 | Poland |
| FJ938288.1 | AJ (Belarus) | KF577586.1 | AJ (Lithuania) | MG952833.1 | Poland |
| GU320211.1 | AJ | KF577588.1 | AJ (Poland) | MG952834.1 | Poland |
| GU361771.1 | AJ (Ukraine) | KR491936.1 | AJ (USA) | MG952835.1 | Poland |
| GU390313.1 | AJ | KX422605.1 | AJ (Ukraine) | MG952836.1 | Poland |
| GU723693.1 | AJ (USA) | KX679399.1 | AJ (Crimea) | MH415452.1 | Poland |
| HM015668.1 | AJ | KX681447.1 | AJ (Poland) | MH542434.1 | Poland |
| HM054510.1 | AJ (USA) | KX686743.1 | AJ (Poland) | MH841892.1 | Poland |
| HM159445.1 | AJ (Ukraine) | KX690094.1 | AJ (Moldova) | MK040987.1 | Poland |
| HM347598.1 | AJ (Belarus) | KX690095.1 | AJ (Czechoslovakia) | MK175430.1 | Poland |
| HM627319.1 | AJ (Lithuania) | KX690209.1 | AJ (Belarus) | MK890795.1 | Poland |
| HQ267514.1 | AJ (USA) | KX702230.1 | AJ (Ukraine) | MK930532.1 | Poland |
| HQ663853.1 | AJ | KX711887.1 | AJ (USA) |  | |
| HQ667591.1 | AJ (Hungary) | KX784496.1 | AJ (Poland) |  |  |
| HQ730608.1 | AJ (Ukraine) | KX786651.1 | AJ (Lithuania) |  |  |
| JF790201.1 | AJ (Ukraine) | KX788167.1 | AJ (Poland) |  |  |
| JF812166.1 | AJ | KX789084.1 | AJ (Austria) |  |  |
| JN204423.1 | AJ (Poland) | KX809696.1 | AJ (England) |  |  |
| JN990448.1 | AJ (USA) | KX833061.1 | AJ (Germany) |  |  |
| JQ228861.1 | AJ (Russia) | KX833062.1 | AJ (Ukraine) |  |  |
| JQ701818.1 | AJ | KX833065.1 | AJ (Lithuania) |  |  |
| JQ701904.1 | AJ | KX834328.1 | AJ (Poland) |  |  |
| JQ702509.1 | AJ | KX842079.1 | AJ (Romania) |  |  |
| JQ702933.1 | AJ | KX844614.1 | AJ (Poland) |  |  |
| JQ703137.1 | AJ | KX881968.1 | AJ (Austria) |  |  |
| JQ703179.1 | AJ | KX946875.1 | AJ (Poland) |  |  |
| JQ703268.1 | AJ | KX953877.1 | AJ (Lithuania) |  |  |
| JQ703313.1 | AJ | KY077676.1 | AJ |  |  |
| JQ703478.1 | AJ | KY353087.1 | AJ (Belarus) |  |  |
| JQ703601.1 | AJ | MG831750.1 | AJ (Ukraine) |  |  |
| JQ703655.1 | AJ | MH817478.1 | AJ (Czech Rep.) |  |  |
| JQ703788.1 | AJ | PopSet 1208984603 | Poland |  |  |
| JQ704081.1 | AJ | PopSet 1530719388 | Poland |  |  |
| JQ704110.1 | AJ | MG952781.1 | Poland |  |  |
| JQ704133.1 | AJ | MG952782.1 | Poland |  |  |

Table S6: GenBank numbers of mitogenomes used for phylogenetic analysis of Poles and Ashkenazi Jews (*15,16*).

| **Individual** | **Haplotype consisting of the following Y-STR markers**  **DYS576-DYS389I-DYS635-DYS389II-DYS460-DYS458-DYS19-YGATAH4-DYS448- DYS391-DYS456-DYS390-DYS438-DYS392-DYS570-DYS437-DYS385a/b-DYS449- DYS393-DYS439-DYS481-DYS533** | **Haplogroup estimation (NevGen)** | **Y-SNP chosen for sequencing** |
| --- | --- | --- | --- |
| S1 | 19-13-24-29-11-17-14-13-19-10-15-24-12-13-17-15-11/14-29-13-13-21-12 | R1b | M269 |
| S2 | 15-13-NA-NA-NA-14-16-NA-20-10-NA-25-11-14-20-NA-11/14-30-NA-10-23-NA | R1a | CTS6 |
| S3 | 17-12-21-29-11-16-15-12-22-10-16-22-10-11-18-16-12/16-29-13-11-22-9 | G2a | P15 |
| S4 | 17-13-23-30-10-19-13-11-20-NA-17-24-10-11-17-14-17/18-32-13-12-25-10 | E1b1b | M123 |
| S5 | 20-13-21-30-11-17.2-14-11-21-10-15-23-NA-11-18-14-13/15-25-12-13-NA-11 | J1a | P58 |
| S6 | 15-14-24-NA-9-18-15-NA-21-NA-17-23-10-NA-16-16-13/16-NA-13-11-19-10 | G2b | L183 |
| S7 | 20-13-21-30-11-17.2-14-11-22-10-15-23-10-NA-18-14-13/15-NA-12-12-24-NA | J1a | P58 |
| S8 | 19-12-22-29-11-16-14-12-20-10-15-24-10-11-19-14-17/19-33-14-12-22-12 | E1b1b | M78 |
| S9 | 20-13-21-30-11-17.2-14-11-21-10-15-23-10-11-18-14-13/15-26-12-12-23-11 | J1a | P58 |
| S10 | 17-13-20-31-10-17.2-14-11-20-10-15-23-10-11-17-14-13/17-26-12-11-25-11 | J1a | P58 |

Table S7: Y-STR haplotypes and corresponding haplogroup estimations.

| **Individual** | **SNP, position (GRCh38)** | **Primer F** | **Primer R** | **Size [bp]** | **Ancestral>**  **Derived allele** | **Sample allele** | **Haplogroup** |
| --- | --- | --- | --- | --- | --- | --- | --- |
| S1 | M269_chrY:  20577481-20577481 | GGATTCTGTTACATGGTATCACAATAGAAGG | CCAAGGTGCTGGGATTACACG | 164 | T>C | C | R-M269 |
| S2 | CTS6_chrY:  2789308-2789308 | GGTTGCTAAGGACTGGATGAAAGAG | TGTTGTGCAGCCATCACCTC | 118 | T>C | C | R-CTS6 |
| S3 | P15_chrY:  21082140-21082140 | TCCTCACATGAATAGAGCCAATGC | GAGAGCCTCAATCCATCATGCA | 193 | C>T | T | G-P15 |
| S4 | M123_chrY:  19602700-19602700 | GGTGTTGCCCAGGAATTTGCAT | TCTTTTCTAACACAGAGCAAGTGACTC | 101 | C>T | T | E-M123 |
| S5 | P58_chrY:  12365936-12365936 | CAGGAGGCCATAATGCAACTAATTCTGA | CTCCCCATGAGGAATGCAGCT | 241 | T>C | C | J-P58 |
| S6 | L183_chrY:  8599095-8599095 | AGCACACCAGATCAAATTCCAGTC | GGTTGGGTCCTGCTACAGGAG | 137 | G>C | C | G-L183 |
| S7 | P58_chrY:  12365936-12365936 | CAGGAGGCCATAATGCAACTAATTCTGA | CTCCCCATGAGGAATGCAGCT | 241 | T>C | C | J-P58 |
| S8 | M78_chrY:  19731417-19731417 | CAGTGGTTTCTGCATTACTCCGTA | GAGCTATAGTGTTCCTTCACCTTTCC | 182 | C>T | T | E-M78 |
| S9 | P58_chrY:  12365936-12365936 | CAGGAGGCCATAATGCAACTAATTCTGA | CTCCCCATGAGGAATGCAGCT | 241 | T>C | C | J-P58 |
| S10 | P58_chrY:  12365936-12365936 | CAGGAGGCCATAATGCAACTAATTCTGA | CTCCCCATGAGGAATGCAGCT | 241 | T>C | C | J-58 |

Table S8: Summary of the Y-SNP-sequencing results.
