# Supplementary material for: Genetic and phylogeographic evidence for Jewish Holocaust victims at the Sobibór death camp
Source: Genome Biol. 2021 Aug 6;22:200. doi: 10.1186/s13059-021-02420-0 (PMC8343952; doi:10.1186/s13059-021-02420-0)
Supplement: Supplementary file 2 — Additional file 2. Review history [file 13059_2021_2420_MOESM2_ESM.docx]

Review History

**First round of review**

**Reviewer 1**

**Are you able to assess all statistics in the manuscript, including the appropriateness of statistical tests used?**

There are no statistics in the manuscript.

**Comments to author:**

This manuscript is one of the most unusual I've reviewed, and at the same time important and fascinating. It is generally a great development that we can use the tools of modern genetic research to bring closure to those individuals brutally murdered during the holocaust. As long as the genetic analysis is solid (which it mostly is; but see below), I believe the paper could be published. On a personal note, every single ancestor of mine was either murdered or is a holocaust survivor, making this paper particularly meaningful to me.

I have a few specific comments.

*	Ethics. It is probably known to the authors (and hinted in the supplement), that it is forbidden by Jewish rabbinical law (which was practiced by the vast majority of Jews who were murdered during the holocaust) to exhume corpses for virtually any purpose, including for DNA testing (which involves an irreversible damage to the skeleton). In this paper, the skeletons were discovered during an excavation, and DNA testing was performed under the assumption that these individuals were not Jewish. But I think it is very important to mention this point explicitly and discuss it early in the main text. [I note that recent rabbinical ruling has permitted (for skeletons discovered in excavations) to use detached teeth for DNA testing. However, it seems that here not only teeth were used.]

Finally, given the identification of these individuals as Ashkenazi Jewish, it is of utmost importance (based on Jewish rabbinical law) to re-bury their skeletons as soon as the research is concluded. This should also be mentioned in the main text.

*	Strength of the evidence. My major scientific comment is regarding the designation of the individuals as Ashkenazi Jews based only on mitochondrial DNA and Y chromosome analyses. These analyses are reasonably convincing (e.g., virtually every single carrier of the K1a1b1a haplogroup studied to date was Jewish; and a perfect match to an Ashkenazi Jew from a database in convincing), but nevertheless, the evidence remains indirect. Also, the strength of the evidence varies substantially across individuals. I am thus wondering why not simply send these DNA sample to microarray genotyping, which will resolve the question with absolute certainty. I am aware that these are low-quality samples, but many ancient DNA labs nowadays have the ability to genotype such samples (and even much older samples) on microarrays and at a very low cost. Standard PCA can then be used to determine whether these individuals had Ashkenazi ancestry. I think the paper could still be published as is, but it would leave uncomfortable doubts on whether the conclusions hold with absolute certainty.

*	Phrasing. A number of sentences in the abstract made me uncomfortable. (1) I think the first sentence should be modified to "Six million European Jews were killed", rather than saying that "it was reported" that Jews were killed. I don't think there's any doubt about this, and as is the sentence can be perceived as offensive. (2) The last sentence ends with "…may act as proof against the Holocaust denial". I think the authors should not be presenting this as a proof against "Holocaust denial". Presenting the genetic evidence in this manuscript as a proof that the holocaust happened can be perceived as very offensive, implying that holocaust denial is a legitimate view and that scientific papers should be written to settle the debate. There's a good reason why holocaust denial is a criminal offense in multiple countries. It may be better to rewrite the last sentence of the abstract as follows (or similarly): "the presented genetic examinations indicate that the remains are of Ashkenazi Jews murdered as part of the mass extermination of European Jews by the Nazi regime, most likely during secret Operation Reinhardt, and not of otherwise hypothesized later non-Jewish partisan combatants."

**Reviewer 2**

**Are you able to assess all statistics in the manuscript, including the appropriateness of statistical tests used?**

There are no statistics in the manuscript.

**Comments to author:**

This manuscript represents the first report of genetic analysis of possible victims of Nazi death camps, and seeks to solve a riddle as to the origin of a set of skeletons found during the excavation of the Sobibor death camp. As such, it is of broad interest to the scientific community and the general public. Unfortunately, the results are only suggestive, insofar as only forensic markers of classical mtDNA and Y chromosome haplogroups were genotyped. Of the 10 individuals genotyped, it is reported that many of the observed haplogroups are much more common in the Ashkenazi Jewish (AJ) population than in the general (non-AJ) European population. However, there are no statistical tests presented to determine the likelihood of this pattern by chance. Moreover, the mtDNA and Y data are presented separately, so it is not clear whether both datapoints are supportive of AJ origin for any of the individuals, or if any of the individuals appear to be very likely non-AJ. It is not clear if there is sufficient DNA material available to perform genomewide genotyping using contemporary DNA microarray technology, but availability of autosomal markers (even if relatively low-resolution) would permit definitive classification of the samples as AJ or non-AJ in origin.

**Authors Response**

**Point-by-point responses to the reviewers’ comments:**

Reviewer #1: This manuscript is one of the most unusual I've reviewed, and at the same time important and fascinating. It is generally a great development that we can use the tools of modern genetic research to bring closure to those individuals brutally murdered during the holocaust. As long as the genetic analysis is solid (which it mostly is; but see below), I believe the paper could be published. On a personal note, every single ancestor of mine was either murdered or is a holocaust survivor, making this paper particularly meaningful to me.

*Response:*

*We would like to sincerely thank Reviewer 1 for his/her critical but supportive comments! We are deeply moved by the cruel fate of the Reviewer’s ancestors. At the same time, we are appreciative to receiving comments not only from a scientific expert, but also from a person, whose opinion on our work is based on compassionate grounds.*

*Further below, the Reviewer suggests language changes, which we are more than happy to follow. Moreover, we would be thankful to receive further language improvements should these be necessary, as we are striving at avoiding any misunderstandings that may arise due to ambiguous language on this extremely sensitive topic.*

I have a few specific comments.

* Ethics. It is probably known to the authors (and hinted in the supplement), that it is forbidden by Jewish rabbinical law (which was practiced by the vast majority of Jews who were murdered during the holocaust) to exhume corpses for virtually any purpose, including for DNA testing (which involves an irreversible damage to the skeleton). In this paper, the skeletons were discovered during an excavation, and DNA testing was performed under the assumption that these individuals were not Jewish. But I think it is very important to mention this point explicitly and discuss it early in the main text. [I note that recent rabbinical ruling has permitted (for skeletons discovered in excavations) to use detached teeth for DNA testing. However, it seems that here not only teeth were used.]

Finally, given the identification of these individuals as Ashkenazi Jewish, it is of utmost importance (based on Jewish rabbinical law) to re-bury their skeletons as soon as the research is concluded. This should also be mentioned in the main text.

*Response:*

*We are particularly thankful for pointing this out and we have adapted the abstract and the second paragraph accordingly. As the Reviewer indicates, we have not been aware of the fact that the remains could have been of Jewish origin, but assumed that they belonged to victims of the Communist and Nazi totalitarian regimes in Poland. Once the Institute of National Remembrance in Lublin received the complex forensic expertise, including the results of the genetic testing, the prosecutor leading the investigation ordered a reburial of the remains at the place of their discovery. The ceremony was accompanied by a local Rabbi and performed according to the Jewish rite. We added this explanation to the manuscript in the abstract and at the end of the main text.*

* Strength of the evidence. My major scientific comment is regarding the designation of the individuals as Ashkenazi Jews based only on mitochondrial DNA and Y chromosome analyses. These analyses are reasonably convincing (e.g., virtually every single carrier of the K1a1b1a haplogroup studied to date was Jewish; and a perfect match to an Ashkenazi Jew from a database in convincing), but nevertheless, the evidence remains indirect. Also, the strength of the evidence varies substantially across individuals. I am thus wondering why not simply send these DNA sample to microarray genotyping, which will resolve the question with absolute certainty. I am aware that these are low-quality samples, but many ancient DNA labs nowadays have the ability to genotype such samples (and even much older samples) on microarrays and at a very low cost. Standard PCA can then be used to determine whether these individuals had Ashkenazi ancestry. I think the paper could still be published as is, but it would leave uncomfortable doubts on whether the conclusions hold with absolute certainty.

*Response:*

*We entirely agree with the reviewer’s position, the evidence that we provide is indirect, but still convincing. We also added – per suggestion of Reviewer 2 – more statistical evidence to our results by providing a quantitative assessment of the findings (see below).*

*We note that the quality of the biological material used in the study was very poor. The remains were badly preserved, even for bones from the time of the Second World War. Microarray genotyping would not have been a viable approach. We have now included verbiage to the text and a table showing the quantitation values (Table S2).*

*We needed to use up all the DNA extracts to be able to produce satisfying mtDNA and Y-STR results from the severely degraded specimens. Since the remains were reburied as soon as the genetic results suggested their Jewish origin, we have no biological material available for any further testing*

* Phrasing. A number of sentences in the abstract made me uncomfortable. (1) I think the first sentence should be modified to "Six million European Jews were killed", rather than saying that "it was reported" that Jews were killed. I don't think there's any doubt about this, and as is the sentence can be perceived as offensive. (2) The last sentence ends with "…may act as proof against the Holocaust denial". I think the authors should not be presenting this as a proof against "Holocaust denial". Presenting the genetic evidence in this manuscript as a proof that the holocaust happened can be perceived as very offensive, implying that holocaust denial is a legitimate view and that scientific papers should be written to settle the debate. There's a good reason why holocaust denial is a criminal offense in multiple countries. It may be better to rewrite the last sentence of the abstract as follows (or similarly): "the presented genetic examinations indicate that the remains are of Ashkenazi Jews murdered as part of the mass extermination of European Jews by the Nazi regime, most likely during secret Operation Reinhardt, and not of otherwise hypothesized later non-Jewish partisan combatants."

*Response:*

*Thank you! We changed the language of the abstract and the end of the manuscript according to the Reviewer’s suggestion.*

Reviewer #2: This manuscript represents the first report of genetic analysis of possible victims of Nazi death camps, and seeks to solve a riddle as to the origin of a set of skeletons found during the excavation of the Sobibor death camp. As such, it is of broad interest to the scientific community and the general public. Unfortunately, the results are only suggestive, insofar as only forensic markers of classical mtDNA and Y chromosome haplogroups were genotyped. Of the 10 individuals genotyped, it is reported that many of the observed haplogroups are much more common in the Ashkenazi Jewish (AJ) population than in the general (non-AJ) European population. However, there are no statistical tests presented to determine the likelihood of this pattern by chance. Moreover, the mtDNA and Y data are presented separately, so it is not clear whether both datapoints are supportive of AJ origin for any of the individuals, or if any of the individuals appear to be very likely non-AJ.

It is not clear if there is sufficient DNA material available to perform genomewide genotyping using contemporary DNA microarray technology, but availability of autosomal markers (even if relatively low-resolution) would permit definitive classification of the samples as AJ or non-AJ in origin.

*Response:*

*We appreciate the Reviewers’ concerns. Since the biological material was severely degraded, we needed to use up the DNA extracts to be able to achieve satisfactory results for Y and mtDNA. We included verbiage and a Table (Table S2) that shows the quantitation values. These would have been far too low for microarray technology targeting nuclear DNA.*

*After the results became known, the remains were reburied according to Jewish rite (see also response to Reviewer 1), which is why there is no more sample available for further genetic analyses.*

*According to religious law, Jewish identity can be considered based on different dimensions including ancestry. Some Judaism movements accept both matrilineal and patrilineal descent as well as conversion, but Orthodox and Conservative Judaism deem a person to be Jewish only, if their mother is Jewish. Therefore, we focused on the maternal lineages of the individuals and we performed additional statistical evaluation of the mitochondrial data in order to strengthen the results.*

*For further evaluation of the mtDNA statistics, we put together two vetted mtDNA datasets. One includes a set of 945 Polish mitotypes (as this was the assumed nationality of the remains), the other one consists of 744 Ashkenazi mitotypes from the literature. We then contrasted the probabilities of the unknown mitotypes to occur in either of them. In all ten remains the probability was higher to find the haplotype in the Ashkenazi dataset (new Table S5).*

*Since the Y-chromosomal evidence is present in Y-STR haplotypes, we did not expect to find similar observations to mtDNA in Y-chromosomal databases, such as the YHRD (https://yhrd.org/). Also, in contrast to mtDNA, there is almost no data available from Ashkenazi Y-chromosomal haplotypes. Therefore, we focused on the frequencies of the established lineages and studied the four individuals assigned to haplogroup J-58, that shared the markers with the Cohen Modal Haplotype, in more detail.*

*Additional changes by the authors*

*• We added information about the reburial of the remains in the abstract and at the end of the main manuscript. We shortened the abstract to comply with the 100-word limit.*

*• We added quantitation results (Table S2) and describe the degraded nature of the samples.*

*• We noticed that the order of the references for the historical supplement and the Figures needed corrections.*

*• Typo in the last name of Felhendler was corrected (the previous version is also used but not in the historical sources we refer to).*

*• In Table S3 one of the GenBank matches was mistakenly called as ‘Unknown’ instead of ‘AJ’, which was corrected.*
